# Long-term forecasting of the impact of EV home charging at different adoption rates on the Egyptian load profile

**DOI:** 10.1038/s41598-025-23647-y

**Published:** 2025-11-10

**Authors:** David Salah Roushdy Beshay, Mohamed Abdul Raouf Shafei, Doaa Khalil Ibrahim

**Affiliations:** Electrical Engineering Department, Faculty of Engineering, Cairo University, Giza, Cairo 12613 Egypt

**Keywords:** Annual energy demand, Bass diffusion model, Electric vehicle (EV), EV market penetration, Long-term forecasting analysis, Load profile, EV uncertainties modeling, Electrical and electronic engineering, Energy grids and networks

## Abstract

Predicting the impact of electric vehicle (EV) fleet charging load on the grid load profile is essential for policymakers during grid planning. A systematic three-stage framework is proposed to forecast the long-term impact of EV home charging on national grids. The framework incorporates: (1) forecasting baseline grid load growth excluding EVs, (2) projecting EV market development, and (3) modeling EV charging behavior uncertainties (plug-in time & rate at home). For the first stage, five models (the autoregressive integrated moving average model, the artificial neural network model based on economic parameters, the nonlinear autoregressive exogenous neural network model, the long short-term memory network, and the convolutional neural network) are evaluated to select the most suitable model. The second stage is investigated using the Bass diffusion model in five penetration scenarios (10%-50%). The third stage is assessed using a probabilistic model based on data acquired by a public survey. The study applied Egypt as a case study, and the results are analyzed using peak load and load factor. Results revealed that 50% market penetration will increase peak load by 20.36% and reduce the load factor by 14.34% by 2040. However, the 10% market penetration limits these impacts to 3.16% and 2.46%, respectively. The study recommends applying demand-side management programs or controlling market expansion to balance the grid demand profile and EV adoption as policy implications. The framework is designed to accommodate a specific area, a city, or a country, as a scalable tool for policymakers addressing the energy-transport nexus in developing economies.

## Introduction

### Motivation and background

In recent years, global attention to Electric Vehicles (EVs) has significantly increased as most countries try to achieve more efficient transportation systems. Accordingly, the penetration rate of EVs has increased in many countries worldwide^1^. However, unmonitored market penetration and uncontrolled charging of EVs lead to negative impacts on the power grids, such as phase imbalance, severe voltage drops, transformer overloading, and a shortage between the supply and the demand energy^2^. The negative influences are more critical in developing markets like the Egyptian market, in which the number of EVs has doubled in 2023 and is expected to increase exponentially in the next few years, as will be revealed in this study. Hence, long-term forecasting of the impact of EV charging load on the utility grid will provide policymakers with valuable insights to take the necessary actions to accommodate the high penetration rates of the EV market.

### Literature review and research gap

Nowadays, there is an excessive research interest in forecasting and modeling the impact of EV charging. For example, the influence of EV charging load is assessed in^3^ when the charging schedule is optimized by a genetic algorithm through two approaches, with and without vehicle-to-grid application. The study applied Texas as a case study. It has considered the future charging load increase until 2030, depending on published statistical data. The impact of charging load on microgrids is also investigated in^4^, taking Okinawa Island as a case study. The study assumed the expected EVs by 500,000 and modeled the charging uncertainties using probability density functions. Besides, it proposed several charging scheduling approaches to minimize the load factor (LF) using genetic algorithm optimization. The EV charging load of parking lots at Caltech was forecasted in^5^. The study employed a long short-term memory network to forecast demand power and day-ahead market price, aiming to increase the profit of parking lot owners. A deep learning framework is also formulated in^6^ to optimize the charging scheduling for a day-ahead period. The primary objective of^6^ is to utilize public charging in parking lots using the Caltech charging sessions dataset and the German energy market dataset as a case study. It modelled the charging uncertainties using a Copula generative adversarial network and forecasted day-ahead charging prices using a time series multi-input nonlinear autoregressive neural network. Finally, it optimised the charging schedule by implementing grey wolf optimization.

The main objective of this article is to model the EV charging load in Egypt and integrate it with the grid load profile. This target will be achieved by predicting the grid load profile excluding the charging load, estimating the expected number of EVs, and modeling the charging uncertainties. In fact, several research studies were conducted to emulate some EV uncertainties via existing historical data sets, whether these sets are of EV or non-EV owners. These emulations may consider developing the EV market penetration over a certain period. In^7^, the Markov process was employed to predict the behavior of EV owners according to the performance and behavior of a region in the United States. It assumed two charging powers while supposing different penetration levels, from 3% to 100%, in a simulation analysis including 200 residential households. When evaluating the impact of EVs on the low-voltage distribution grid in the study of^8^, most of the uncertainties that occur due to the random behavior of the owners were considered. The introduced model assumed various arrival times of each EV at home, the tendency of plugging in the EV at different values for the state of charge (SOC) based on the average daily distance an owner usually travels, different charging durations based on SOC whenever it is plugged in, and finally considered two different charging scenarios. The first scenario utilizes the 3.7 kW single-phase charger for the whole fleet, and the second one uses the 11 kW three-phase charger for the overall fleet. The K nearest neighbor (KNN) probabilistic model was integrated with the geographic information system in^9^, and used to make a probabilistic model that forecasts the demand load of the EV uncontrolled charging by predicting the behavior uncertainties of owners. These predictions are based on historical data recorded by the Australian authorities. EVI-Pro software was applied to predict the charging load pattern of EVs based on GPS historical data in the study of^10^, and the penetration level of EVs was projected to be 30% by 2030. The study assumed two charging scenarios (home-dominant and work-dominant charging scenarios) and considered several charging powers. However, all EVs whose SOC is less than 100% are supposed to charge daily. In^11^, the recorded data of a survey, including owners of 1600 EVs and 1400 households, is utilized to conduct an empirical study. The study considered the given data at a specific time without considering the future expansion of the EV charging demand load or the progress of the grid demand load. By^12^, the EV fleet of the Chinese Shenzhen was divided into four categories based on the EV application (private vehicles, taxis, buses, and official vehicles) to facilitate modeling the EV uncertainties and forecasting EV market penetration. The EV behavior of the four categories is predicted using a probabilistic model based on the data acquired by a public survey, and the penetration levels through seven years were modeled using the Bass model. However, the study did not account for the development of the typical demand load over the projected five years. The impact of the charging load on a medium voltage network is assessed in^13^. It applied the Casablanca, Morocco, as a case study. It investigated two charging scenarios (day charging at work and charging at home arrival) and accounted for some of the charging uncertainties based on statistical reports. However, the study did not consider any development of the grid demand load or the charging load. Moreover, it only assumed the 3.7 kW charger. Six EV penetration levels are examined on two systems (IEEE-33 and IEEE-69 bus) in^14^. It accounted for the SOC of the EV using a probability distribution function. However, it failed to study other uncertainties, such as varying charging starting times, plug-in rates, and charger types. Also, it did not consider the development of the grid demand load or the charging load.

Table 1 summarizes some published studies^7–14^ regarding the applied system, the developed load, and EV uncertainties. In the last row, the features of this article are presented regarding the same items.

To the best of the authors’ knowledge, forecasting the EV impact in Egypt is rarely discussed in the literature. The number of public charging stations and the status of the EV market in Egypt are discussed in^15^. The study listed the number of EVs per type and the kWh price in public stations. The factors affecting the purchase intention of EVs in Egypt are investigated in^16^ by conducting a public survey. It highlighted how governmental policies affect the preference of end users to purchase an EV. The development of the EV market in Egypt from 2021 to 2040 is examined in^17^, and the study investigated the number of EVs and the environmental impacts using a bottom-up stochastic model. It is deduced that the expected number of EVs can reach almost 3.5 million EVs in 2040.

Collectively, the published research studies have advanced the field of evaluating the impact of EVs in several networks over a certain period and considering EV uncertainties. Yet, some research limitations or gaps can be outlined as follows:

- There is a notable lack of combining the development of the grid load during the prediction process in several studies. However, it is essential when predicting the impact of the EV charging load, especially for grid planning and policy considerations.
- As the impact of the EV charging load depends on the number of EVs, investigating the development of the EV market over the study duration is very significant, which is not considered in all studies.
- While several studies addressed the prediction of the impact of EV charging load in various contexts worldwide, there is a shortage of studies addressing this research point in Egypt.

Therefore, this study aims to bridge these gaps by developing a holistic framework that integrates the long-term forecasting of the baseline grid load, the development of the EV market, and a detailed probabilistic model of user charging behavior, applied to the under-researched Egyptian context.

**Table 1:** Summary of the literature review and the contribution of this study.

Study	System applied		EV load expansion		EV uncertainties			
	Grid level / network	Grid load development	EV penetration rate	EV Market Development	Charger types	Battery capacity used	Charging start time	Plug-in rate at home
^7^ 2018	Distribution Grid/ Network in the USA	N/C	6 levels considered	N/C	Two types, not simultaneously	Constant every day	Variable based on the Markov process	N/C
^8^ 2019	Distribution Grid/ Network in Denmark	N/C	5 levels considered	N/C	Two types, not simultaneously	Variable – statistical reports	Variable – statistical reports	Considered
^9^ 2020	National Grid/ Network of Australia	N/C	11 levels considered	N/C	One type	Variable – KNN probabilistic model	Variable – KNN probabilistic model	N/C
^10^ 2021	Distribution Grid/ 10 studied feeders	N/C	One level considered	N/C	Two types simultaneously	Variable – historical data	Variable – historical data	N/C
^11^ 2021	National Grid/Network of Arizona	N/C	One level considered	N/C	Two types, not simultaneously	Variable – historical data	Variable – historical data	N/C
^12^ 2020	National Grid/Network of Shenzhen	N/C	7 levels considered (annual level over 7 years)	1 scenario - using the Bass model	Two types simultaneously	Variable – local survey	Variable – local survey	N/C
^13^ 2024	National Grid/Network of Casablanca	N/C	N/C	N/C	One type	Variable – statistical reports	Variable – statistical reports	N/C
^14^ 2024	Distribution Grid/ IEEE-33 & 69 bus systems	N/C	6 levels considered	N/C	One type	Probability density function	One period assumed	N/C
This article	National Grid/ Egyptian Grid	Considered -integrated with several EV penetration scenarios		Considered – via 5 scenarios using the Bass model	Two types simultaneously	Considered - using a probabilistic model via a local survey		

N/C: Not considered.

### Paper contributions and organization

In this study, a comprehensive framework is formulated to carry out a long-term forecasting study of the impact of the EV market penetration in Egypt and its influence on the daily load curve up to 2040 while considering the aforementioned research gaps . The forecasting will be separated into three main stages: the development of the energy demand load excluding the EV charging load, the development of the EV market penetration, and the EV fleet charging characteristics. The first stage is achieved using five models to decide the most suitable one for the Egyptian energy demand. Subsequently, the second stage is done using the Bass diffusion model. Lastly, the third stage is processed using a probabilistic model based on the public survey results to account for most EV charging uncertainties.

The results of this study provide robust forecasting for the EV market in Egypt and its impact on the Egyptian daily load curve while considering the usual development of the energy demand load and the development of the EV market penetration over the study duration. Such forecasts in the early stages of the EV market penetration can provide policymakers with valuable insights into what to expect in case of uncontrolled EV market penetration or uncontrolled charging, and help them take crucial actions to maintain a secure energy market while developing a more environmentally friendly transportation market.

The rest of this study is organized as follows: the Egyptian case study is described in Sect. Case study description, the methodology of the applied framework over its three stages is presented in Sect. Methodology of the applied comprehensive framework, and the five applied models for forecasting annual energy demand are fully described in Sect. First stage: forecasting annual energy demand. Forecasting the EV market penetration is introduced in Sect. Second stage: Forecasting EV market penetration, and the prediction of the EV charging uncertainties is demonstrated in Sect. Third stage: predicting EV owners’ behavior. The results of applying the designed framework to the Egyptian market to forecast the impact of EV penetration are presented in Sect. Forecasting results for the impact of the EV market, including possible policy implications and the limitations of the proposed framework. The conclusions and suggested future work are highlighted in Sect. Conclusions and future work.

## Case study description

The study presents a systematic framework for predicting the long-term impact of EV home charging loads on the grid load profile, with the Egyptian market serving as a case study.

### Electricity pricing structure

Electricity pricing in Egypt is separated into three sectors: residential, commercial, and industrial. The EV charging in Egypt is either home charging or public charging. Home charging is a residential load that follows a graduated tariff system. It means that the kWh price increases with higher monthly consumption. On the other hand, the pricing of public charging stations in Egypt is not yet fully standardized; however, in most cases, the kWh price is based on the required charging power.

As discussed in^11,18^, home charging represents 80% of EV charging; however, the public charging that presents only 20% may cause a severe impact on the grid due to the high charging rates in public charging stations^19^. Regarding Egypt, the number of public charging stations is limited, while the Egyptian vision is to accommodate around 800,000 slow-charging units (home charging units) in 2040^17^. Therefore, only home charging is considered in this study, while public charging is ignored.

Until now, no demand-side management (DSM) programs have been implemented in Egypt for the residential sector. It means that neither time-of-use pricing nor real-time pricing is considered in-home charging.

###  EV fleet

Usually, EV fleets are categorized as private, official, taxis, and buses^12^. Concerning Egypt, the latter three categories are minor, representing only 5% of total EV fleets. Accordingly, the available data regarding these categories is very limited in Egypt and hard to collect through a public survey. On the contrary, the private EV fleet is increasing rapidly and considerably, as will be displayed in Sect. Second stage: Forecasting EV market penetration of this study. Also, this increase aligns with the country’s policy towards expanding this market in Egypt. Accordingly, only the private EV fleet is considered in this study. The government’s vision for the EV market is to increase the market to 30% in 2030 and 50% in 2040^17^.

## Methodology of the applied comprehensive framework

**Fig. 1:**
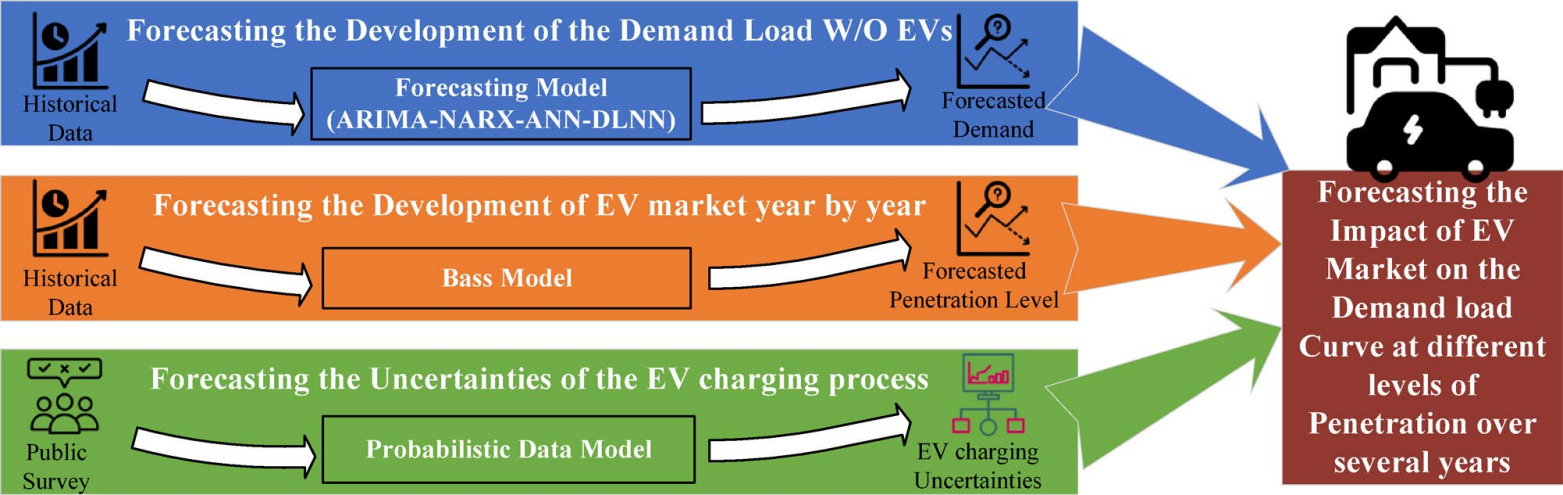
Schematic diagram for the proposed framework.

In this study, long-term forecasting analysis for the impact of EV market penetration in Egypt will be investigated through the upcoming years up to 2040. The proposed framework is illustrated in the schematic diagram shown in Fig. 1.

1. At first, the total annual energy demand of the Egyptian grid will be predicted (excluding the EV fleet charging load) using five models to select the most suitable one:
  - Autoregressive Integrated Moving Average (ARIMA) model^20^,
  - Nonlinear Autoregressive Exogenous (NARX) neural network model^21^,
  - Artificial Neural Network (ANN) model based on economic parameters^21^.
  - Multivariate Deep Learning Neural Networks (DLNN):
    i. Long Short-Term Memory (LSTM) model,
    ii. Convolutional Neural Network (CNN) model.
2. Afterwards, the EV market penetration is forecasted using the Bass diffusion model to predict the number of EVs in the Egyptian market.
3. Finally, a public survey is conducted to predict the behavior of EV owners during the charging process.

In other words, the integration of phases 2 and 3 comprises the future EV charging load profile, while phase 1 covers the projection of the demand load separately (excluding the EV fleet charging load). So, the results from phases 2 and 3 are added to phase 1 to get the projection of the grid load, including the EV charging demand load for a certain duration.

For the investigated case study of Egypt, the forecasted annual energy demand will be used to simulate the load curve of the national Egyptian grid on the day of maximum demand each year up to 2040. Then, predicting the EV market penetration alongside the expectancy of behavior for EV owners will provide the forecasted impact of the EV market on the daily load curve each year.

### First stage: forecasting annual energy demand

The historical data used in this section is between 1982 and 2023, with 42 data points. This data series of the Egyptian annual energy demand is collected based on the annual reports of the Egyptian Electricity Holding Company^22^. These reports are published yearly in the middle of the following year (e.g., the report of 2023 was published in June 2024). Accordingly, the historical data available is up to 2023, and the forecast in this study starts from 2024 to 2040.

The goal is long-term forecasting for the electrical energy demand in Egypt over a definite duration. Forecasting energy demand can be approached as a time series analysis^23,24^ or as a nonlinear relation between the energy and correlated economic parameters^23,25,26^. Accordingly, well-established models that are extensively used in literature are applied in this study for the same task:

1. ARIMA, as a conventional time-series analysis^23,24,27^.
2. NARX network, as a nonlinear time-series analysis (recurrent neural network architecture)^25–27^.
3. ANN depends on economic parameters as a nonlinear relation between the energy demand and the correlated economic factors such as gross domestic product (GDP), population, and temperature^24–26^.
4. Multivariate deep learning models, based on the economic factors mentioned before (multivariate recurrent neural networks)^28^. The LSTM network and CNN are applied^5^.

Although the ARIMA model is a relatively older statistical method compared to newer approaches in time series forecasting, it is still extensively used in the literature for long-term forecasting as a baseline model for its proven reliability in time series forecasting^2,29^. Moreover, ANN architecture is not a cutting-edge model, but its application in long-term forecasting based on economic parameters is still implemented in recent applications and research areas^23,24^. On the other hand, deep learning models require large data sets to operate properly^30^. In the tested case, the available historical data is limited. Accordingly, the economic factors are utilized as predictors for the deep learning networks to compensate for this shortcoming. Also, the networks are designed to avoid overfitting that might occur due to the limited historical data (as will be shown later in this section).

Consequently, this study will use five models with the four approaches mentioned above to compensate for the limited available historical data. Then, the results will be appraised to choose whichever is better in forecasting the energy demand in the base case^23,27,31^, and the output of this stage will be considered the baseline demand load of the proposed framework.

#### ARIMA model

**Fig. 2:**
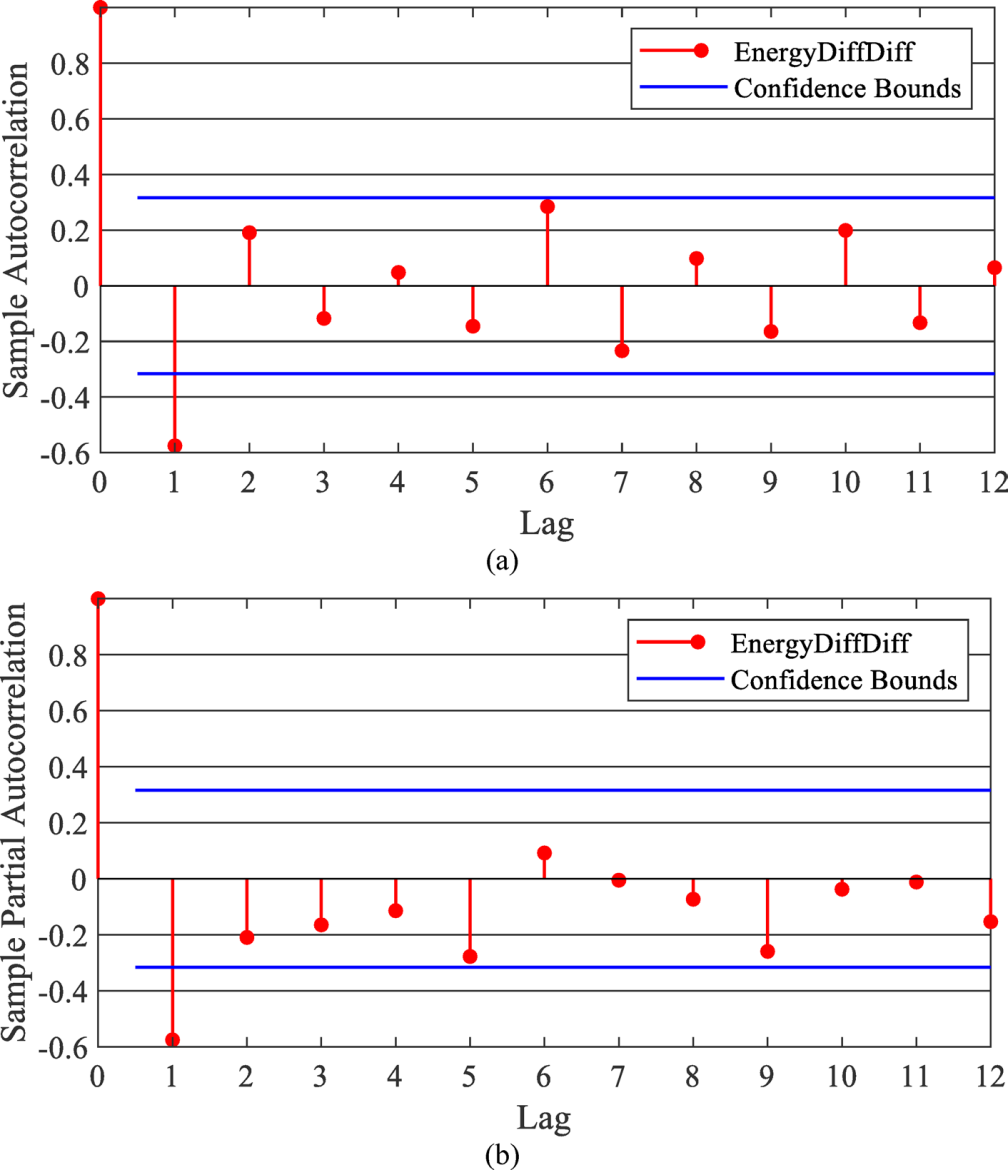
ACF and PACF correlograms for the time series of annual energy demand.

ARIMA modeling is one of the most used models for long-term load forecasting in the literature^32^. The Box-Jenkins method provides a step-by-step methodology for forming a robust ARIMA model^20^. The model order ($\:p$, $\:d$, $\:q$) should best fit the time series, where $\:p$ denotes the autoregressive terms, $\:d$ is the differences needed for stationarity, and $\:q$ describes the number of lagged forecast errors in the prediction equation. The following steps are conducted using the Econometrics Toolbox in MATLAB software to determine the model order and validate it:

- The first step is to test for stationarity. Using the KPSS (Kwiatkowski–Phillips–Schmidt–Shin) tests^33^, the time series is stationary after the second difference.
- The second step is to analyze the correlogram of the Autocorrelation Function (ACF) and the Partial Autocorrelation Function (PACF) of the second difference of the time series^21,34,35^. As revealed in Fig. 2, the significance of the ACF drops below the confidence bounds after the first lag, which means that the moving average order is 1. Besides, the significance of the PACF drops under the confidence bounds after the first lag, which means that the auto-regressive order is 1. Accordingly, the model will be ARIMA (1, 2, 1) based on the KPSS test, ACF, and PACF.
- The third step is to validate the developed ARIMA model, as will be conducted in Sect. Evaluating the five generated models.

#### NARX network

NARX network is an artificial neural network architecture that uses past values of a time series $\:y\left(t\right)$ as an input, as well as an external time series input $\:x\left(t\right)$ to forecast future values of the time series $\:y\left(t\right)$^27,31,36^.

**Fig. 3:**
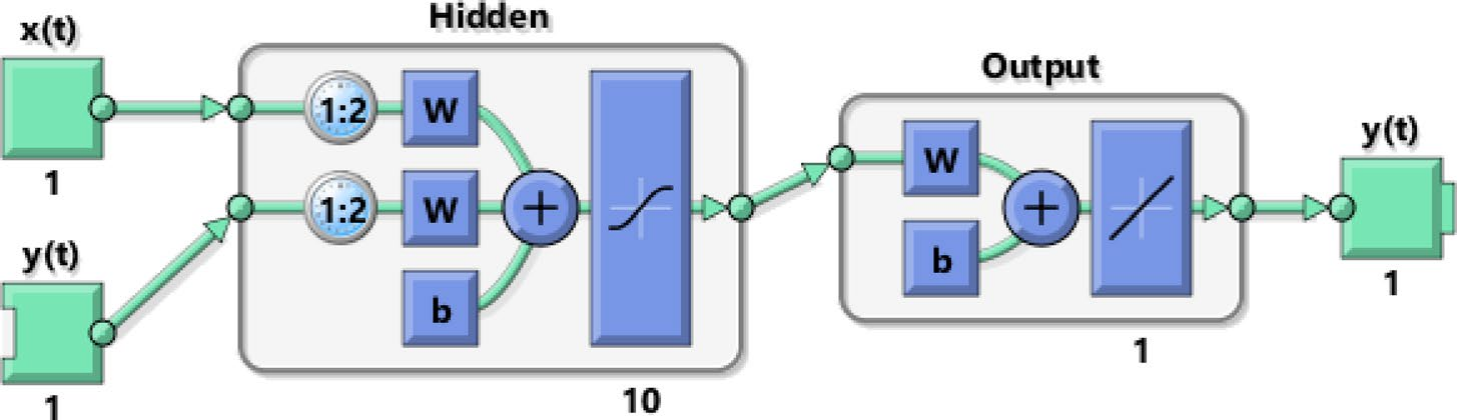
NARX network architecture in the open-loop during training mode.

The external input $\:x\left(t\right)$ is set to be fixed time steps from 1 to 42, and the response $\:y\left(t\right)$ is the energy time series. The model also utilizes $\:y\left(t\right)$ with a time delay of 2 as the second input. As shown in Fig. 3, the network consists of 3 layers: the input layer, the output layer, and the hidden layer (which consists of 10 nodes).

**Fig. 4:**
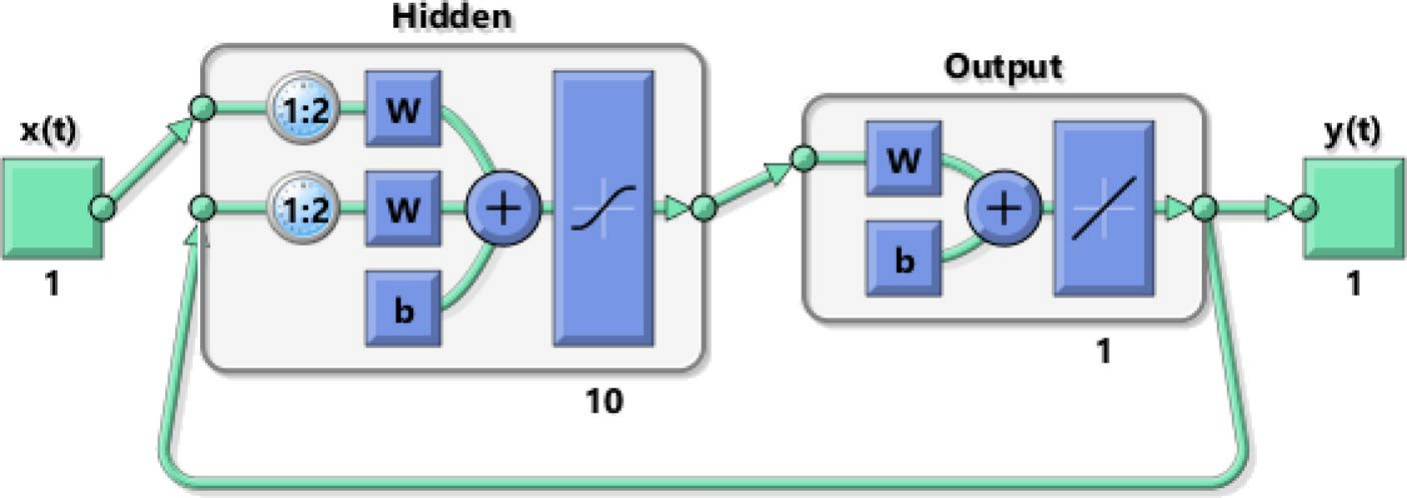
NARX network architecture in closed-loop during forecasting mode.

The data is divided into a 70% training set, a 15% validation set, and a 15% testing set. The Levenberg-Marquardt back-propagation procedure is applied as the most common training algorithm in NARX networks^31,37^. As displayed in Fig. 3, the network is trained initially in the open-loop configuration using MATLAB neural network time series toolbox. Then, it is transformed to the closed-loop configuration^27^, as shown in Fig. 4, to forecast future values. Finally, the results are validated, as presented in Sect. Evaluating the five generated models.

#### ANN based on economic parameters

This model is constructed via an ANN network based on economic parameters as inputs^23^. The network is trained to obtain the relation between the annual energy demand and some economic parameters^38^. The most used parameters in literature are the weather, population, and GDP^26,31,37,39^. In this study, the proposed economic parameters are the population, GDP, GDP per capita, the annual mean temperature, and the 5-year smoothed mean temperature. Each of these parameters includes 42 data points related to the identical duration of the historical data of the annual energy demand (between 1982 and 2023) and generated from the World Bank group data.

The first step is to examine the correlation between the annual energy demand and the economic parameters to ensure that these parameters are correlated to the annual energy demand. Besides, the GDP and the GDP per capita are compared, and the annual mean temperature is evaluated against the 5-year smoother mean temperature. This is done using the Pearson correlation coefficient $\:\rho\:(A,\:B)$ between two variables $\:A$ and $\:B\:$for the column length parameter of $\:N$, which is presented by^31,40^:

1 $$\:\rho\:(A,B)=\frac{1}{N-1}\:\sum\:_{i=1}^{N}\left(\frac{{A}_{i}-{\mu\:}_{A}}{{\sigma\:}_{A}}\right)\left(\frac{{B}_{i}-{\mu\:}_{B}}{{\sigma\:}_{B}}\right)$$

Where, $\:{\mu\:}_{A}$, $\:{\mu\:}_{B}$ denote the mean values of the two variables $\:A$, and $\:B$, respectively, and $\:{\sigma\:}_{A}$, $\:{\sigma\:}_{B}$ describe their standard deviation values. Accordingly, the correlation coefficient $\:\rho\:(A,B)$ value will be evaluated to judge the correlation strength between the two random variables based on Table 2^40^.

**Table 2:** Strength of the correlation between two random variables.

Value of correlation coefficient	Strength of the correlation
0.90 to 1.00	Very high correlation
0.70 to 0.89	High correlation
0.50 to 0.69	Moderate correlation
0.30 to 0.49	Low correlation
0.00 to 0.29	Little to no correlation

The achieved results of the correlation study between the input parameters and the energy demand are tabulated in Table 3. It ensures that the correlation between the energy demand and all input parameters falls in the “very high correlation region” except for the annual mean temperature, which falls in the “high correlation region”. Nonetheless, the correlation with the GDP per capita is slightly stronger than the GDP. Also, the correlation with the 5-year smoothed mean temperature is stronger than the annual mean temperature. Consequently, the economic parameters that will be used to train the ANN network are as follows: Population (Po), GDP per capita (G), and the 5-year smoothed mean temperature (T).

**Table 3:** The correlation coefficient between the annual energy demand and the economic parameters.

Economic parameters	Strength of the correlation with annual energy demand
Population (Po)	0.9859 (very high correlation)
GDP	0.9468 (very high correlation)
GDP per capita (G)	0.9497 (very high correlation)
Annual mean temperature	0.8137 (high correlation)
5-year smoothed mean temperature (T)	0.9549 (very high correlation)

**Fig. 5:**
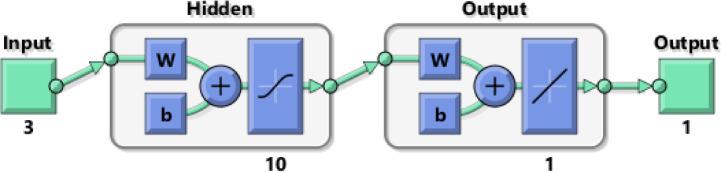
ANN network based on three economic parameters (Po-G-T).

The ANN network displayed in Fig. 5 comprises three layers: input, hidden, and output layers. These layers consist of three nodes (Po-G-T), 10 nodes, and a single node, respectively. The network is trained using the Levenberg-Marquardt back-propagation procedure in the MATLAB neural network fitting toolbox. Then, the input time series (Po-G-T) is forecasted using one of the time series models. In this study, the ARIMA model is applied. So, these forecasts can be used as inputs to the trained ANN network to predict the energy.

#### Two multivariate DLNN models

Several deep learning networks have been developed for time-series forecasting. Deep learning networks need huge input data sets to perform correctly^28,30^. However, the available dataset in this study, as described earlier, is too limited to be used as training data for a deep learning network. This limited data set will cause network overfitting. To mitigate this shortcoming, the economic parameters described in Sect. ANN based on economic parameters are utilized as predictors along with the historical data of the energy demand. This will increase the size of the input data set and prevent the network from overfitting. In other words, the networks are trained using four predictors: energy demand historical data, Po, GDP, and T. Each predictor consists of 42 historical observations.

**Fig. 6:**
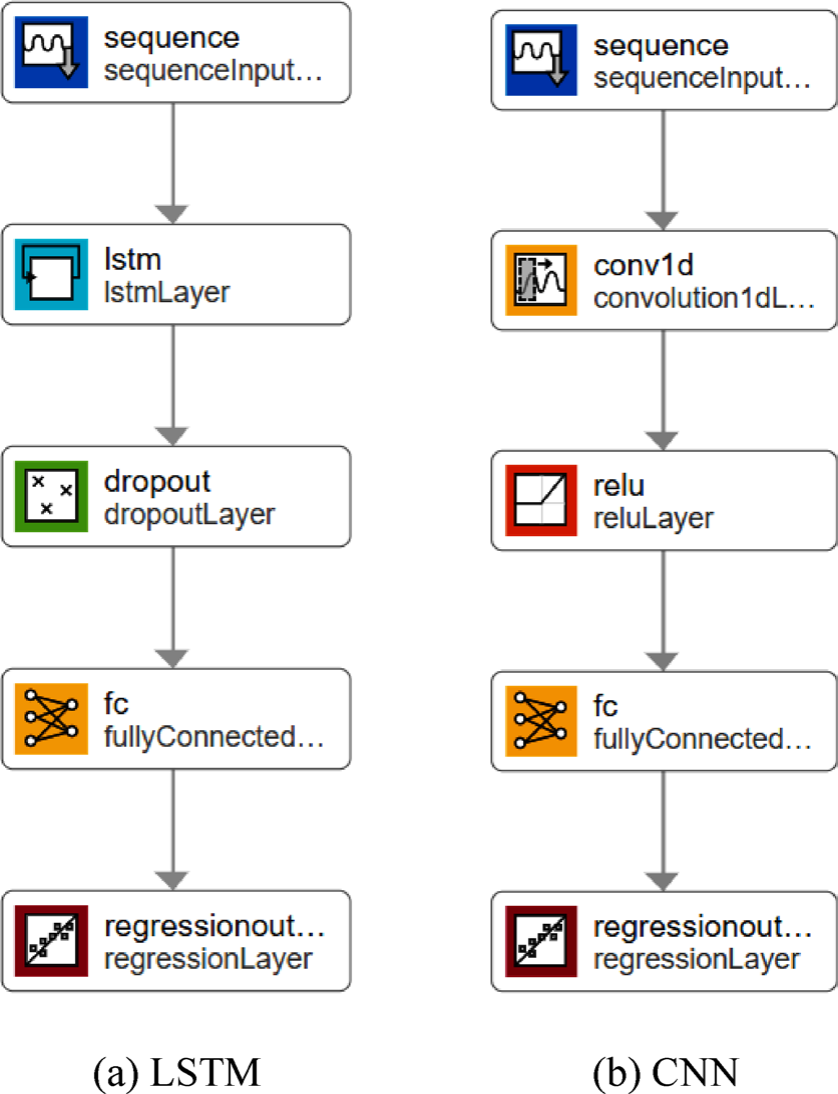
Architecture of applied deep learning networks (LSTM & CNN). (**a**) LSTM, (**b**) CNN.

The proposed framework will apply two different deep learning networks, LSTM and CNN networks. Networks are created and trained using MATLAB. Figure 6 shows the architecture of the networks. Each network consists of five main layers. Both networks start with a sequence input layer of four features, and end in a fully connected layer and a regression layer with one output. The difference between the networks is in the second and third layers. The LSTM network contains a layer with 128 hidden units and a dropout layer, while the CNN network contains a convolution layer of filter size equal to five, filter number equal to 16, and a rectified linear unit layer. Both networks are trained using 200 epochs to avoid overfitting.

#### Evaluating the five generated models

**Fig. 7:**
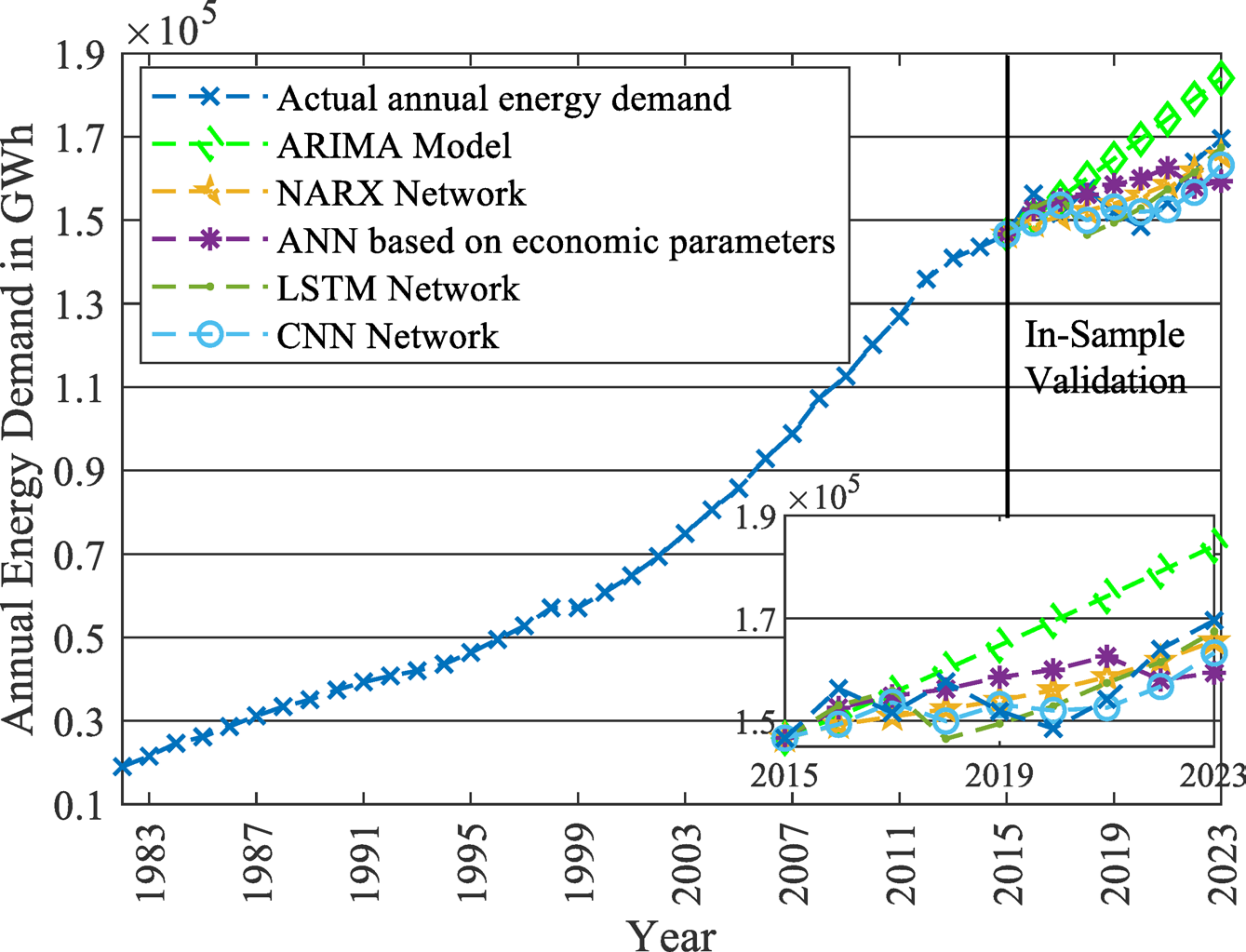
In-sample forecasting (2016–2023) for validating the five implemented models.

Evaluating the five generated models is conducted using in-sample forecasting. In-sample forecasting considers the last 20% of the time series observations (eight observations) as validation data points and predicts the corresponding values by the generated models^21,35^, as revealed in Fig. 7. These predictions are compared to the original validation data points found in the historical data set of the energy demand load using the evaluation criterion. In this study, two criteria are used: Root Mean Square Error (RMSE) and Mean Absolute Percentage Error (MAPE), which are the most widely used evaluation criteria in the literature^21,23,37^.

The mathematical relationship of RMSE and MAPE is given below, where $\:n$ is the total count of observations within the sample, and the forecasted and actual value of the time series at time $\:t$ is described by $\:{F}_{t}$ and $\:{Y}_{t}$, respectively.

**Fig. 8:**
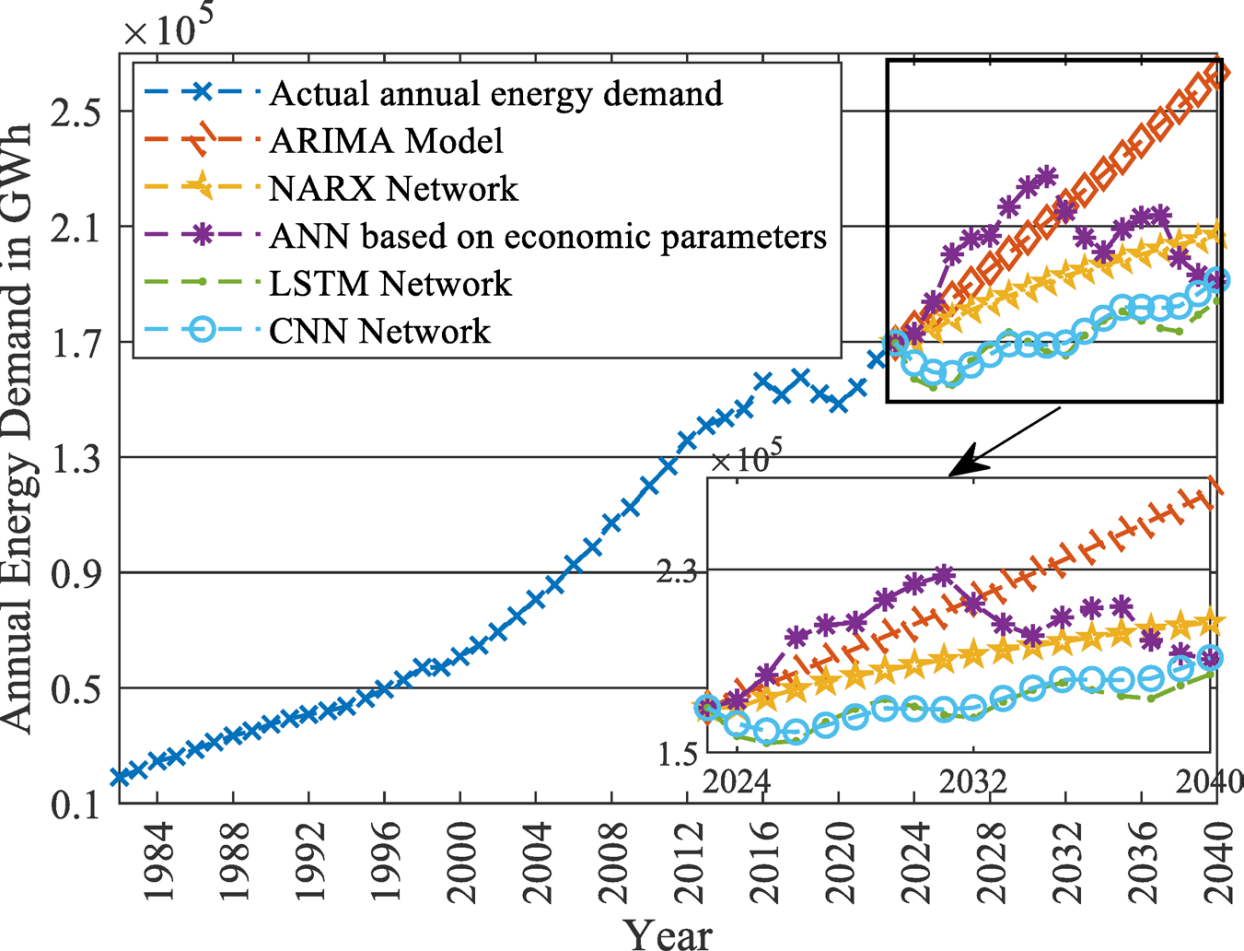
Forecasted annual energy demand up to 2040 based on the five implemented models.

The results of the RMSE and MAPE for the five tested models are summarized in Table 4. The results indicated that the NARX model outperformed the other models, having the least RMSE and MAPE values (4,731 GWh and 2.67%, respectively). The RMSE values of the deep learning networks are the second in line. LSTM and CNN values are higher than those for NARX by only 5.2% and 11%, respectively. The values of the ANN model were nearly 150% of the NARX model (7,177 GWh and 4.08%, respectively). Finally, the ARIMA model values were almost three times those of the NARX model, with values of 13,690 GWh and 7.62%, respectively. It indicates that the NARX model provided the most accurate in-sample forecast. Hence, the forecasting till 2040 in the rest of the study will be based on the NARX model, while the forecasted results from 2024 to 2040 for the five models are displayed in Fig. 8.

**Table 4:** RMSE and MAPE values of each of the five forecasting models.

Model type	RMSE (GWh)	MAPE
ARIMA model	13,690	7.62%
NARX network	4,731	2.67%
ANN based on economic parameters	7,177	4.08%
LSTM	4,981	2.68%
CNN	5,251	2.88%

#### Sensitivities affecting the forecasting models

While the NARX model has the best in-sample performance, it is essential to acknowledge that there are several limitations for long-term forecasting using this model. The forecasting results may be influenced by several correlated factors, e.g., the variation in the provided economic parameters mentioned before. In addition to those factors, the following sensitivities may impact the forecasting results:

- Policy changes, e.g., applying DSM programs or updating pricing structures.
- Economic fluctuations, since considerable changes in economic growth patterns will influence commercial and industrial demand significantly.
- Technological adoption, such as the wide-scale electrification trends in commercial and industrial sectors.
- Consumer behavior shifts, such as embracing energy-efficient devices in residential households or working from home (like during the COVID-19 pandemic).

**Fig. 9:**
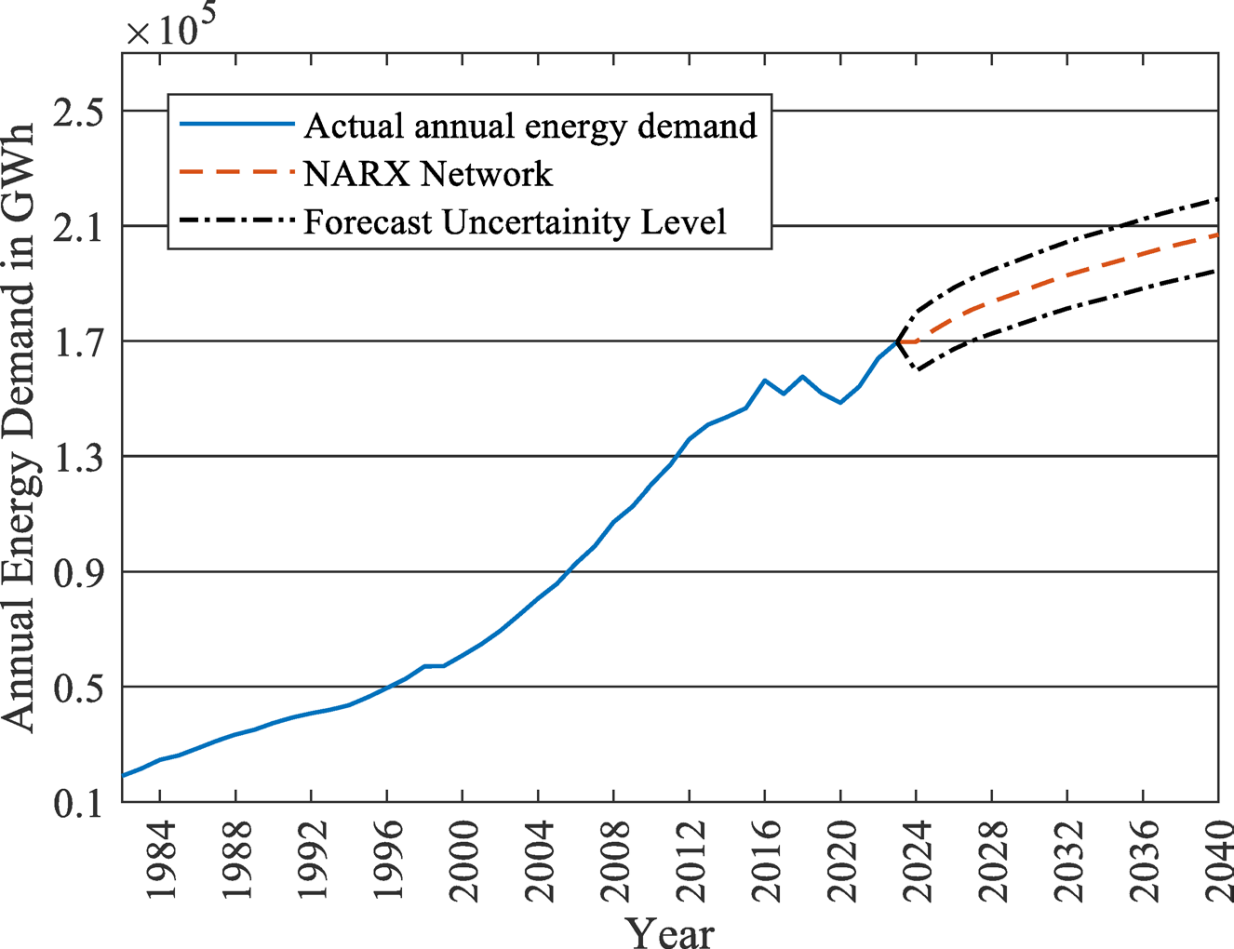
Uncertainty level of the NARX network.

Accordingly, while the NARX model performs well within the in-sample validation period, the underlying dynamics and the relationships between the past energy demand values and time steps may be influenced in the future by such factors. Besides, the limited historical dataset may limit the model’s ability to learn and forecast accurately. This limitation was partially addressed by using multiple forecasting approaches and in-sample validation. However, it remains a consideration for long-term forecasting. To account for these limitations, a Monte Carlo dropout analysis is conducted on the NARX network for 1000 iterations to determine the uncertainty level of the network forecasts. The results revealed that the NARX model has 6% uncertainty level, as shown in Fig. 9. However, for simplicity of analysis, only the mean value of the forecast will be used in the remaining parts of the study.

Hence, it is recommended that the historical data of this framework be updated regularly as new data becomes available. This will allow the model to adapt to emerging trends and maintain the forecasting accuracy over the study horizon.

### Second stage: forecasting EV market penetration

According to the Egyptian Central Agency for Public Mobilization and Statistics (CAPMAS), the total number of licensed vehicles in 2023 was 5.5 million. By doing a straightforward time-series analysis, it is expected to reach six million in 2040. On the other hand, based also on CAPMAS, the number of EVs in Egypt in 2022 was 2,466 and nearly doubled to 4,917 in 2023. Despite the 4917 EVs representing only 0.083% of the total number of vehicles, it designates that the Egyptian EV market is in the innovator stage and will start rapid growth soon^1^.

The Bass diffusion model is one of the most used models for analyzing and forecasting new markets^41,42^. The model formulation is as follows:

4 $$\:N\left(t\right)=m.\frac{1-{e}^{-\left(In+Im\right)t}}{1+\frac{Im}{In}.{e}^{-\left(In+Im\right)t}}$$

The cumulative number of adopters until time $\:t$ is presented by $\:N\left(t\right)$, $\:m$ is the total expected adopters in the market, $\:In$ is the coefficient of innovation (which signifies the independent adoption of innovators), and $\:Im$ is the coefficient of imitation (which denotes the dependent adoption influenced by the innovators).

In this study, five values of $\:m$ will be investigated, starting from 10% and up to 50% (with a 10% step of the total EV market), which is expected to be six million EVs as mentioned before. This will ensure that the study will cover most of the possible market projections up to 2040. The Bass model is applied to forecast the EV market in Egypt up to 2040 in these five scenarios. The historical data provided by CAPMAS regarding the number of licensed EVs in Egypt is used to estimate the $\:In$ and $\:Im$ of the Bass model using a nonlinear Generalized Reduced Gradient (GRG) solver in Excel software. It aims to minimize the squared error between forecasted and historical data. The results of the forecast data compared to the actual historical data are presented in Table 5 for 2020 to 2023.

**Table 5:** Historical data, forecast data for the number of EV adopters, and the RMSE between them.

Year	Actual	Forecasted
2020	190	357
2021	1,019	1,046
2022	2,466	2,376
2023	4,917	4,943
RMSE	96.54	

Due to the limited historical data of EV adoption, a sensitivity analysis was conducted on Bass model parameters ($\:In$ & $\:Im$). The values of $\:In$ and $\:Im$ were intentionally changed by ± 10% while keeping $\:m$ constant at its maximum value 50% to observe the impact on $\:N\left(t\right)$. The impact is analysed at 2030 and 2040 to present the early growth and later saturation phases, respectively.

**Fig. 10:**
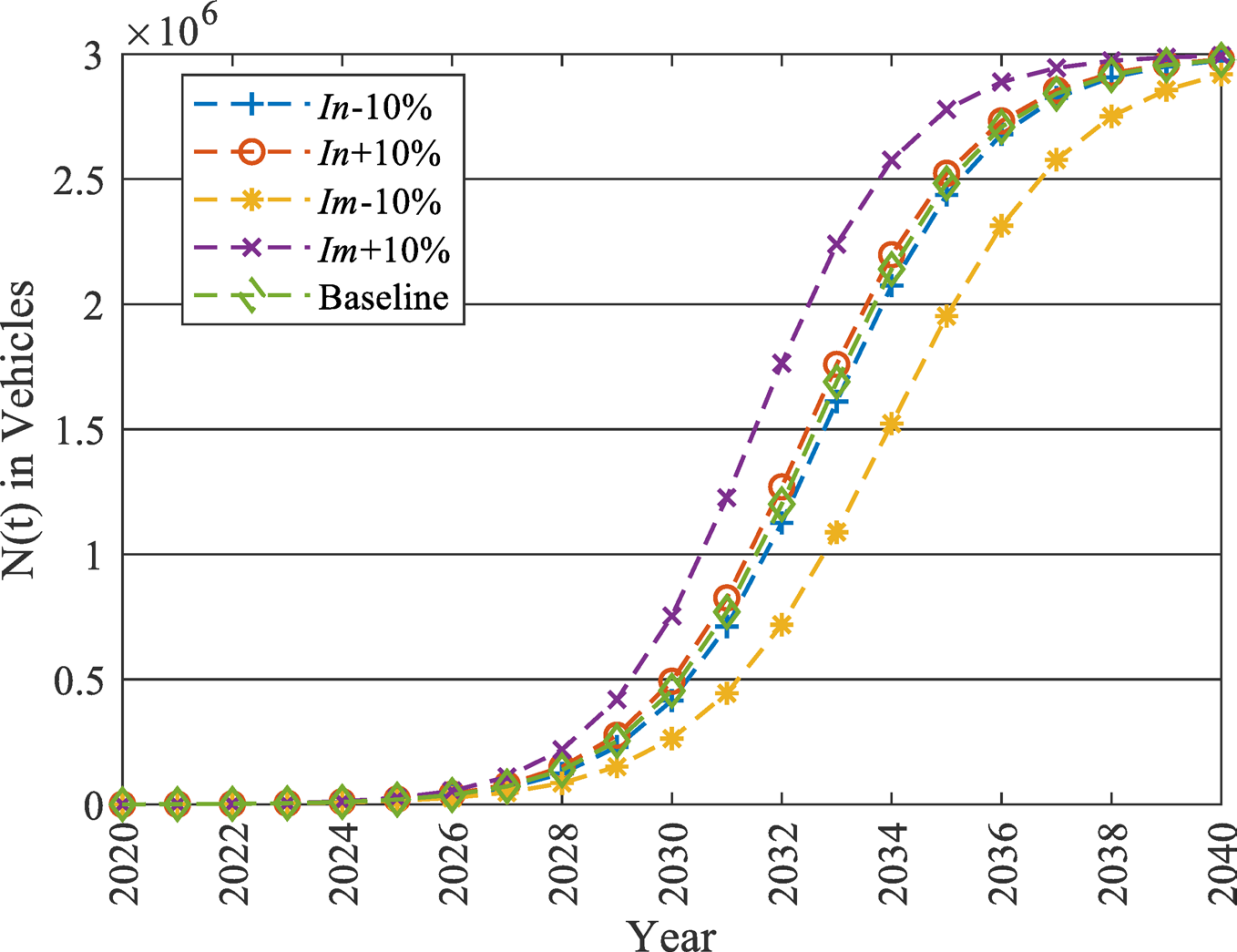
Sensitivity analysis of the Bass model by changing $\:In$ and $\:Im$ by ± 10% while keeping $\:m$ constant at 50%.

As shown in Fig. 10, the model is more sensitive to the changes in $\:Im$ in both phases. Besides, as revealed in Table 6, the model is more sensitive to parameter uncertainty in the early growth phase, when $\:Im$ is increased by 10% it reflects a 65.66% growth in the baseline at 2030, while revealing only a 0.56% increase in 2040. However, Fig. 10 reveals that the parameter uncertainties do not disturb the market development pattern across all scenarios. Accordingly, it can be concluded that despite the values of $\:N\left(t\right)$ may be affected by parameter uncertainities, the market development pattern remains constant.

**Table 6:** Sensitivity analysis of the bass model by changing and by ± 10% while keeping constant at 50%.

Changed parameter	At 2030					At 2040				
	Baseline $\:\varvec{N}\left(2030\right)$	With − 10%		With + 10%		Baseline $\:\varvec{N}\left(2040\right)$	With − 10%		With + 10%	
		$\:\varvec{N}\left(2030\right)$	% change	$\:\varvec{N}\left(2030\right)$	% change		$\:\varvec{N}\left(2040\right)$	% change	$\:\varvec{N}\left(2040\right)$	% change
$\:In$	454,583	415,391	-8.62%	492,610	8.37%	2,977,026	2,974,491	-0.09%	2,979,104	0.07%
$\:Im$	454,583	262,996	-42.15%	753,047	65.66%	2,977,026	2,919,171	-1.94%	2,993,626	0.56%

**Fig. 11:**
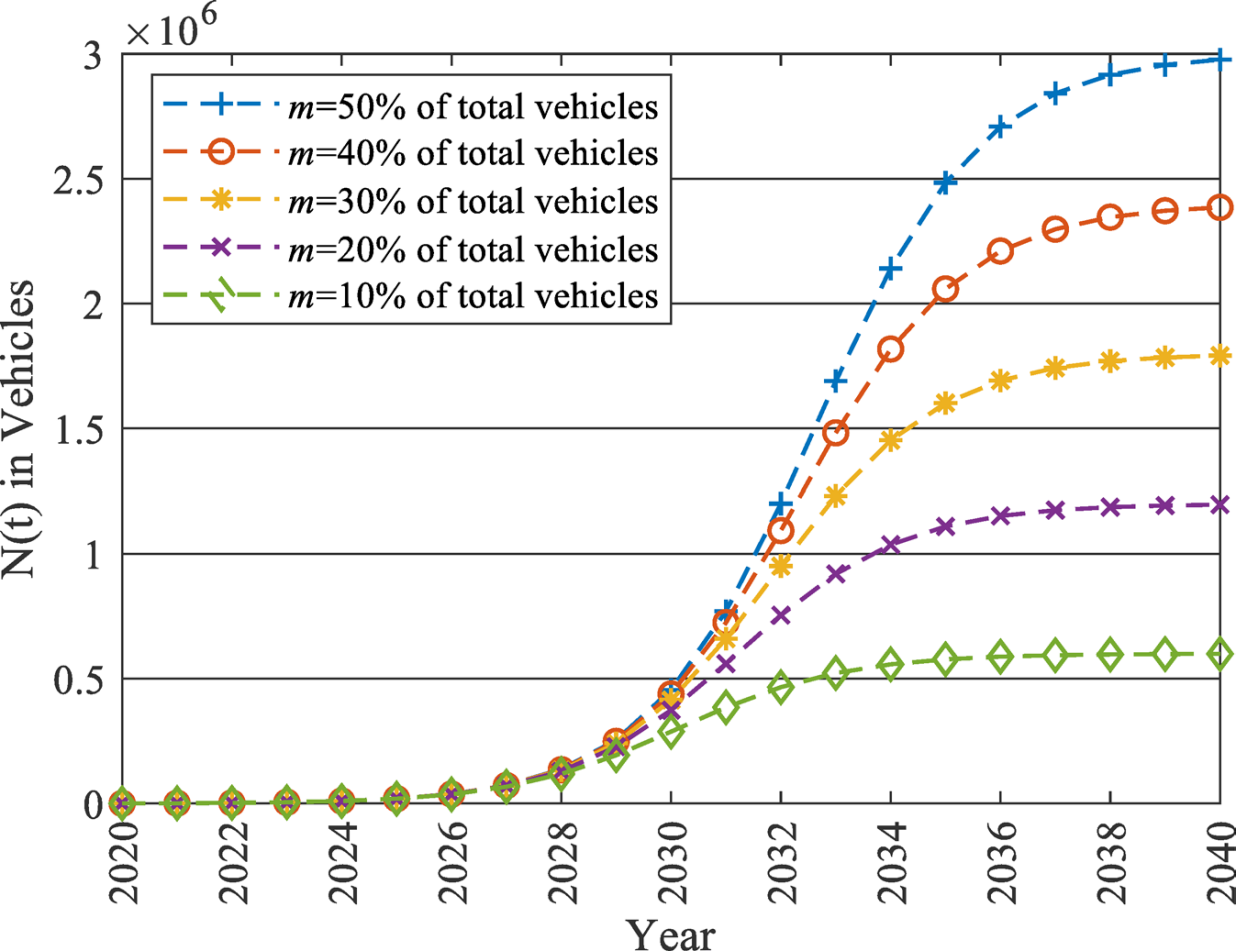
Forecasted cumulative number of EV adopters [N(t)] up to 2040 using the Bass diffusion model.

Finally, the estimated model is used to forecast the $\:N\left(t\right)$ with the baseline values of $\:In$ and $\:Im$ for each of the five scenarios up to 2040, as displayed in Fig. 11. It indicates that the parameter $\:m$ is a critical parameter. By changing its magnitude, the shape of the EV market development is changed significantly. The government can control the development of the EV market by controlling this value and thus the effect on the national grid.

### Third stage: predicting EV owners’ behavior

The EV owners’ behavior, including their driving profile, can significantly affect the expected charging load profile^6^. To accurately simulate the EV owners’ behavior, a public survey was conducted so that the results could be transformed into a model that simulates these behaviors in the form of a charging schedule, as will be shown in Sect.  Probabilistic model formation.

#### Determining the sample size

The first step is to determine the sample size, which is needed to be achieved by the survey. According to^43,44^, the sample size depends on the degree of confidence or accuracy. The required sample size $\:\left(S\right)$ is defined based on^43] and [44^ as follows, respectively:

5 $$\:S=\frac{{X}^{2}NP(1-P)}{{d}^{2}(N-1)}+{X}^{2}P(1-P)$$

6 $$\:S=\frac{N}{1+N.{d}^{2}}$$

Where $\:{X}^{2}$ is the table value of chi-squared for the first degree of freedom at the desired confidence level, $\:N$ is the total population size, $\:P$ is the population proportion, and $\:d$ presents the degree of accuracy that usually equals 0.05.

In this section, the total population is the total number of EV owners, which is assumed to reach three million EVs in the Egyptian market according to the scenario for which $\:m$ equals 50% (as presented in Fig. 11). Using the two mentioned approaches, the sample size tends to be 400 samples. Consequently, the survey aimed to reach 400 responses to ensure a sufficient representation of the total population.

#### Public survey design and implementation

The public survey is designed as an online questionnaire using the Google Forms platform and shared randomly to ensure random sampling. It includes three main sections:

- Personal information (gender, age, geographical location, etc.)
- Vehicle data and driving behavior during a typical working day (type of vehicle, average daily driving distance, average time for home arrival, etc.)
- Tendency to participate in a DSM program if available.

It is conducted over three months to collect 400 responses. The responses were from three Egyptian governments: Cairo, Giza, and Qalyubia, representing 42%, 34%, and 24% of total responses, respectively. The four main factors that will determine the shape of the EV charging load profile are as follows^8^:

- The time the EV connects to the grid throughout the day.
- The duration of the charging process (depending on SOC) when connected to the grid.
- Plug-in rate of EV.
- Charger type (AC or DC charger) and its power.

Thus, the questionnaire targeted the first three factors, while the fourth one was difficult to characterize by the survey due to the limited number of EV owners in Egypt. Meanwhile, the first three factors can be emulated by typical vehicle owners (similar to behavior with EVs). So, the survey targeted both EV and typical vehicle owners. The response from EV owners represents 5% of the total responses, but it does not form a bias since the owners’ behavior regarding these three factors will not differ by vehicle type.

#### Survey results

The responses to the survey were analyzed using Excel to remove duplicates and filter unwanted outliers. Then the responses were grouped considering the three surveyed factors.

##### Time of connection to the grid

**Fig. 12:**
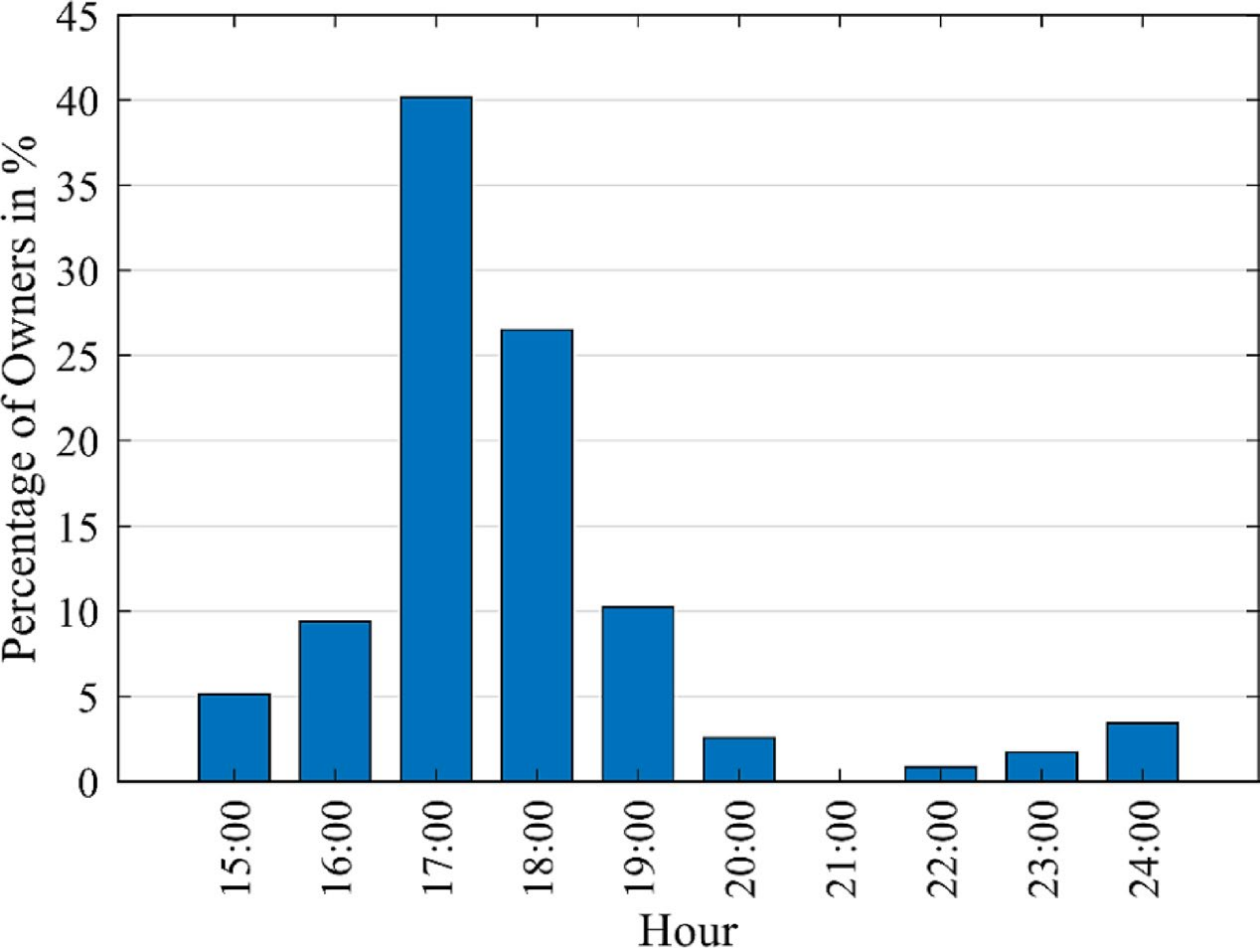
Average arrival time of EV owners at home throughout the day.

EV owners usually connect their cars once they arrive home^45^. Accordingly, the survey targets the arrival time at home, as shown in Fig. 12. It designates the start time of the EV charging at home $\:\left({t}_{start}\right)$. The results show that the most common arrival times are at 16:00, 17:00, 18:00, and 19:00 by 9.4%, 40.17%, 26.5%, and 10.26% of the total number of owners, respectively. The remaining percentage of 13.67% can be neglected as the percentage of EVs during the rest of the hours is much lower.

##### The SOC when connecting to the grid

$\:SOC\:\left(d\right)$ of the EV battery on any day $\:\left(d\right)$ depends on the remaining capacity of the battery $\:Q\left(d\right)$ since the last plug-in at the end of a day $\:\left(d\right)$ and the nominal capacity of the battery ($\:Qn$). In this study, $\:Qn$ is assumed to be fixed and equal to 40 kWh.

7 $$\:SOC\:\left(d\right)=\frac{Q\left(d\right)}{Qn}$$

By the end of a day $\:\left(d\right)$, the remaining capacity of the battery $\:Q\left(d\right)$ depends on the accumulated used $\:Qu\left(d\right)$, since the last plug-in. $\:Qu\left(d\right)$ depends on the driven distance $\:x\left(d\right)$ multiplied by the average EV energy use per km $\:c=0.2\:kWh/km$^18^, and the used capacity till the previous day $\:(d-1)$, as follows:

8 $$\:Q\left(d\right)=Qn-Qu\left(d\right)$$

$$\:Qu\left(d\right)=x\left(d\right)\times\:c+Qu\left(d-1\right)$$

The time when the charging process will end $\:\left({t}_{end}\right)$ depends on $\:{t}_{start}$, $\:Qu\left(d\right)$, charger power $\:{P}_{charger}$, and the charger efficiency $\:\eta\:$, as follows:

9 $$\:{t}_{end}={t}_{start}+\frac{Qu\left(d\right)}{{P}_{charger}\:\times\:\eta\:}$$

**Fig. 13:**
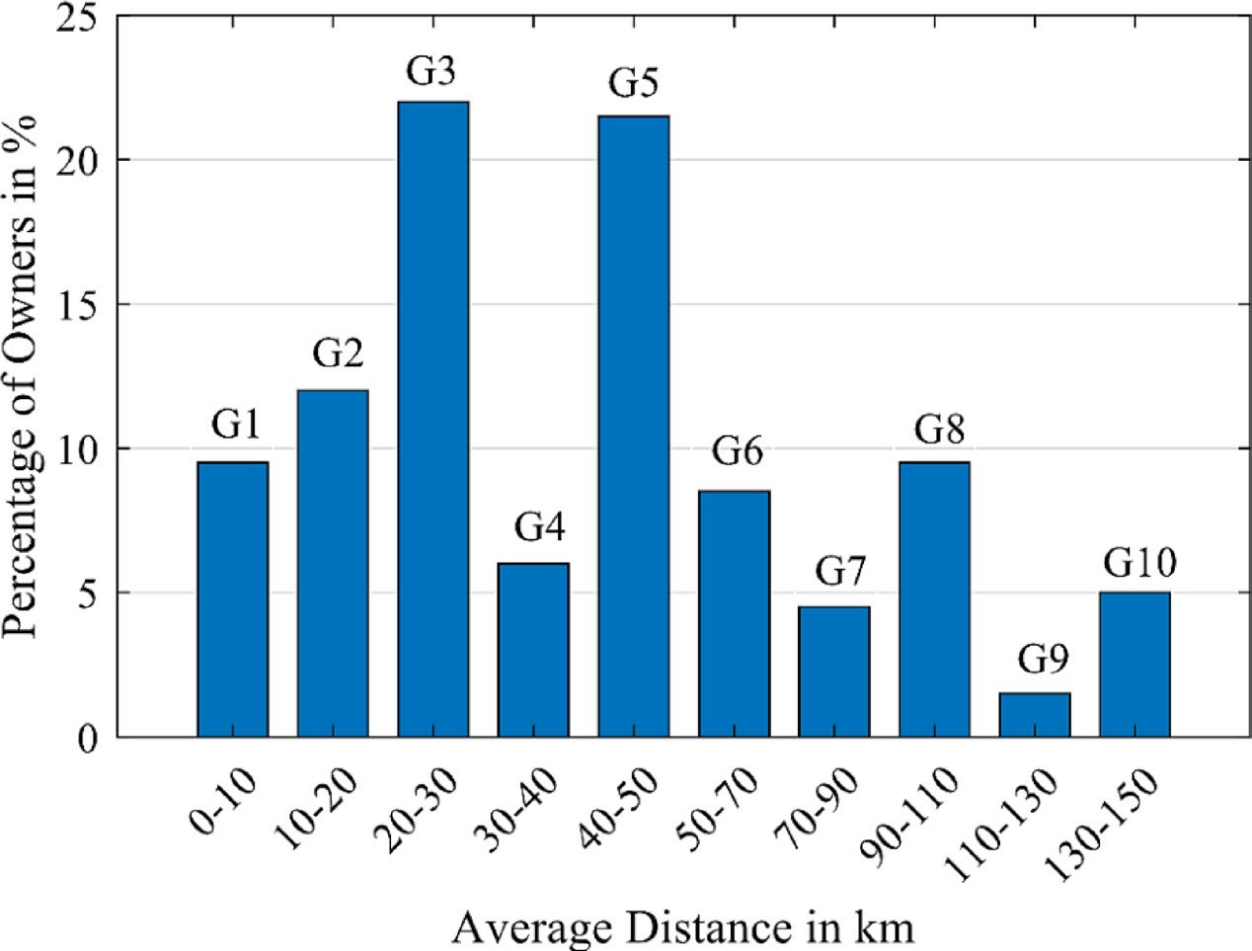
EV owners groups (10 groups) based on the average daily driven distance by an owner.

Based on the conducted survey, the owners are categorized into 10 different groups $\:\left(Gi\right)$ and $\:i=\left(1\:to\:10\right)$ according to the average daily driven distance $\:x\left(d\right)$, as revealed in Fig. 13.

##### Plug-in rate of EV

Finally, the survey showed that there is a correlation between the average distance driven and the plug-in rate of the EV at home. The more the average distance an owner drives daily, the more the probability of plugging in his EV at a higher SOC and vice versa. In other words, if someone drives longer distances daily, they will tend to recharge their EV more often to ensure its availability to cover the lengthy distances they used to. It was very similar to what is stated in^8^.

**Fig. 14:**
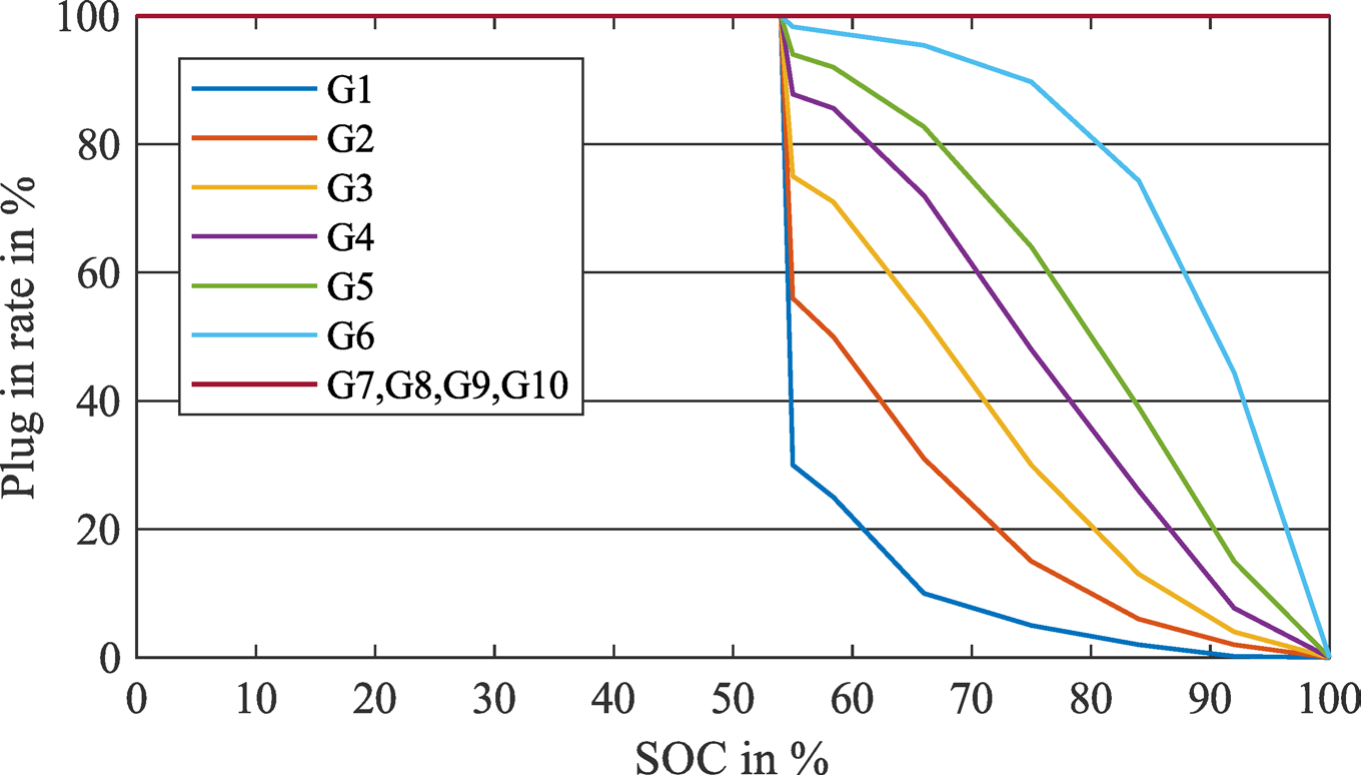
EV plug-in rate at home based on daily average driven distance and SOC at arrival.

These results provide a relation between the plug-in rate at home, the SOC of the EV, and the daily average distance driven by the owner, as displayed in Fig. 14.

#### Probabilistic model formation

In addition to the above factors, some conservative assumptions were made as follows:

- Only home charging was considered in the study, and public stations were ignored as stated in Sect. Electricity pricing structure.
- Also, 80% of home chargers were considered single-phase of 7.4 kW, 32 A, and only 20% were considered three-phase of 11 kW, 16 A^46^. This assignment remains fixed for each EV throughout the simulation iterations, reflecting the permanent installation of home charging equipment. Additionally, the 80:20 distribution is based on market availability and the residential electrical infrastructure in Egypt, where single-phase supply is more prevalent.
- Moreover, charger efficiency was assumed to be constant at 90% for all EVs^47^.

**Fig. 15:**
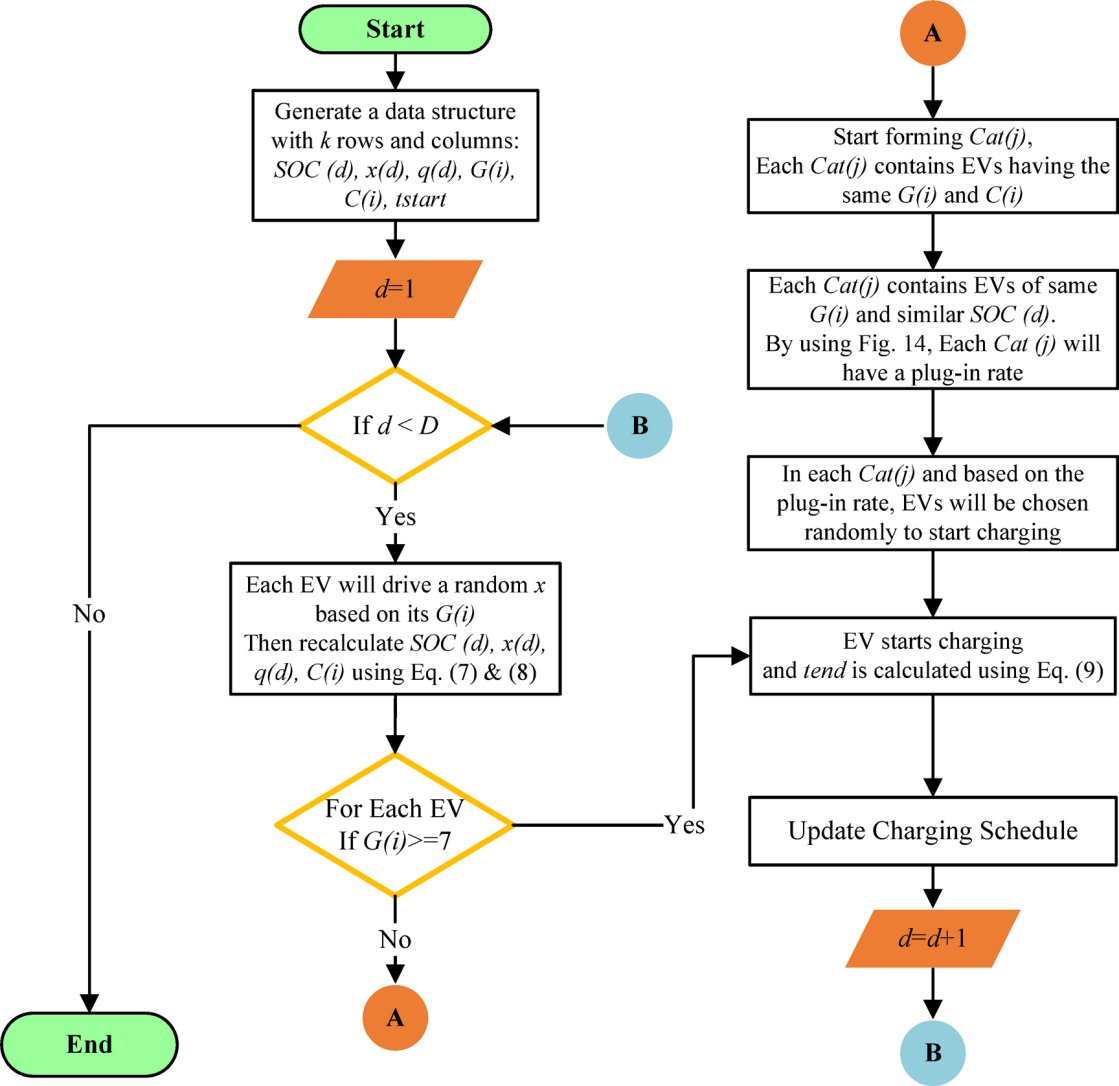
Flowchart of a model based on survey results to predict EV charging schedule over several days.

The model is then formulated using MATLAB software, as per the flowchart shown in Fig. 15, based on the survey results and the mentioned assumptions.

*Step #1* The inputs to the model are the number of EVs ($\:k$) and the number of days ($\:D)$, where $\:D$ is the number of iterations the model will go through.

*Step #2* The model starts by generating a data structure that contains the properties of each EV (e.g. $\:SOC\:\left(d\right)$, $\:x\left(d\right)$, $\:Q\left(d\right)$, $\:G\left(i\right)$, $\:{t}_{start}$…etc.), where the length of the structure equals the number of EVs ($\:k$) and $\:d=1\:to\:D$.

*Step #3* All EVs that have a group number $\:G\left(7\right)$ to $\:G\left(10\right)$ will charge daily (in every iteration) as revealed in Fig. 14.

*Step #4* Each EV will drive a random $\:x\left(d\right)$ based on its $\:G\left(i\right)$, and then the $\:SOC\:\left(d\right)$ will be recalculated according to Eq. (7) and Eq. (8).

*Step #5* A new parameter will be introduced for each EV, which is the class number $\:C\left(i\right)$. $\:C\left(i\right)$ indicates the accumulated driven distance $\:\left(x\right)$ by an EV from the last plug-in until the day $\:\left(d\right)$ according to Table 7, where $\:i=1\:to\:10$.

*Step #6* After each EV gets its $\:C\left(i\right)$; all the EVs remaining from *step #3* are clustered together in categories $\:Cat\left(j\right)$ based on their $\:G\left(i\right)$ and $\:C\left(i\right)$. Each $\:Cat\left(j\right)$ consists of EVs having the same $\:G\left(i\right)$ and the same $\:C\left(i\right)$. Accordingly, all the EVs in the same $\:Cat\left(j\right)$ will have similar $\:SOC\:\left(d\right)$.

*Step #7* Using the $\:G\left(i\right)$ and the $\:SOC\:\left(d\right)$ of each $\:Cat\left(j\right)$, we can generate the plug-in rate of this category using Fig. 14. By plug-in rate, the model will randomly determine the EVs in each $\:Cat\left(j\right)$ that will be plugged-in on the day $\:d$ and the remainder of the EVs will not be plugged in.

*Step #8* The parameters of the EVs that are plugged in will reset: $\:SOC\:\left(d\right)=1$, $\:x=0$, $\:Q\left(d\right)=Qn$. The time $\:{t}_{end}$ is calculated using Eq. (9).

*Step #9* The model will iterate for $\:D$ times by starting from *Step #3* till the end.

**Table 7:** Class number based on the accumulated driven distance since the last plug-in.

$\:\varvec{C}\left(\varvec{i}\right)$	$\:\varvec{x}$	$\:\varvec{C}\left(\varvec{i}\right)$	$\:\varvec{x}$
1	0 < $\:x$ <= 10	6	50 < $\:x$ <= 70
2	10 < $\:x$ <= 20	7	70 < $\:x$ <= 90
3	20 < $\:x$ <= 30	8	90 < $\:x$ <= 110
4	30 < $\:x$ <= 40	9	110 < $\:x$ <= 130
5	40 < $\:x$ <= 50	10	130 < $\:x$ <= 150

After running the model several times equal to the number of days $\:\left(D\right)$, the charging schedule is generated for each iteration, and a sample is given in Table 8. As shown, it consists of four columns. The first column is named the EV index, which indicates the ID of a specific EV in the simulated fleet. The second column is the charging power of the EV. The third and fourth columns indicate whether the EV is charging, and if it is not, and if it is charging, the $\:{t}_{start}$ and $\:{t}_{end}$ for each plugged-in EV is offered on a given day that is chosen from the total number of iterations $\:\left(D\right)$. This data represents the predicted charging behavior (schedule) during a typical working day over the year.

**Table 8:** Sample of the charging schedule output of the EV owner behavior simulation model.

EV index	Charging power (kW)	Charging start time ($\:{\varvec{t}}_{\varvec{s}\varvec{t}\varvec{a}\varvec{r}\varvec{t}})$	Charging end time ($\:{\varvec{t}}_{\varvec{e}\varvec{n}\varvec{d}}$)
1	.	.	.
.	.	.	.
.	.	.	.
112	0	0	0
113	7.4	18:00	20:42
114	7.4	17:00	19:42
115	0	0	0
116	0	0	0
117	0	0	0
118	7.4	18:00	20:06
119	7.4	18:00	20:42
120	7.4	16:00	18:06
121	11	19:00	20:24
122	11	19:00	20:24
123	7.4	17:00	19:06
124	0	0	0
125	0	0	0
126	0	0	0
127	0	0	0
128	0	0	0
129	0	0	0
130	7.4	17:00	19:06
131	7.4	17:00	18:30
132	7.4	17:00	19:42
133	0	0	0
.	.	.	.
.	.	.	.
400	.	.	.

## Forecasting results for the impact of the EV market

As described in the first stage of the applied framework, the Egyptian annual energy demand $\:E\left(y\right)$ is forecasted using the NARX model up to 2040 based on the historical data between 1982 and 2023. Accordingly, the Egyptian daily load curve for any year $\:\left(y\right)$ up to 2040 is estimated using the following equation. It approximates the power increase ($\:Pc)$ as a constant rate, where the total annual energy consumed in the year $\:\left(y\right)$ is expressed by $\:E\left(y\right)$.

10 $$\:Pc=\frac{E\left(y\right)-E(y-1)}{365\:days\times\:24\:hours}$$

**Fig. 16:**
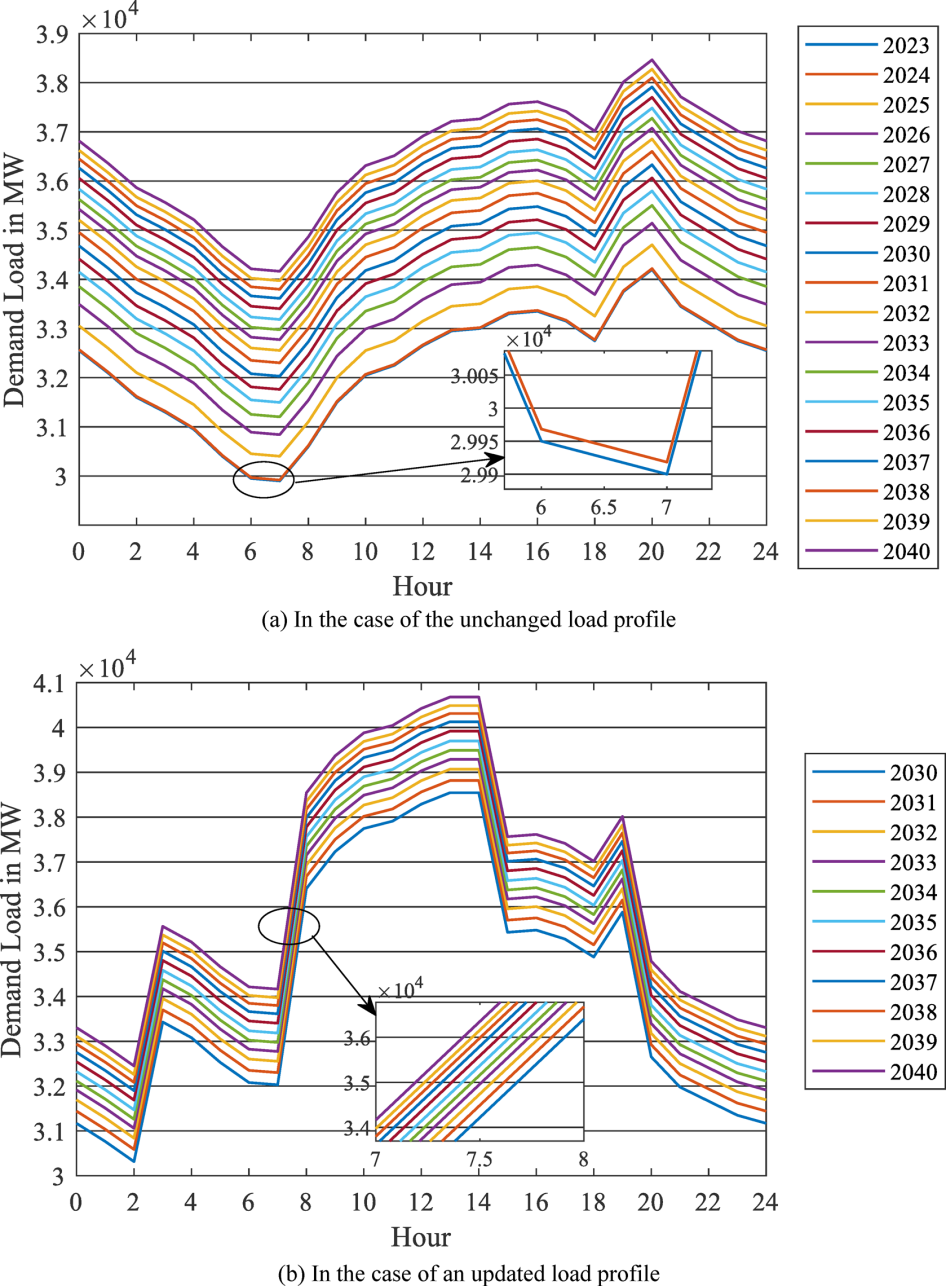
Daily load curve for 2023 up to 2040 based on energy forecasting using the NARX model. (**a**) In the case of the unchanged load profile, (**b**) In the case of an updated load profile.

Based on the actual daily load curve of 2023 provided by the annual reports of the Egyptian Electricity Holding Company^22^, the daily load curves from 2024 to 2040 are estimated by adding the output of Eq. (10). The results are displayed in Fig. 16 (a).

However, the probability that the load profile shape will change over a long period from 2024 to 2040 is very high, especially with renewable energy integration, which can influence the load profile significantly^48^. Therefore, this framework shall be updated on a year-by-year basis to maintain accurate forecasts. A scenario-based analysis is conducted to assess the proposed framework’s robustness to load profile changes. The assumed scenario is based on multiple aspects relating to Egypt’s energy transition goals, as follows:

- The Egyptian vision, in which the renewable energy generation is expected to reach 30% of total energy generation by 2030, and approximately a third of this percentage will be achieved from solar energy^49^, which means that solar power generation will represent 10% of total power demand.
- Solar energy generation is concentrated during daylight hours (from 7:00 to 17:00), creating a surplus in energy generation during midday. Accordingly, this surplus must be balanced by demand shifting to avoid using expensive energy storage solutions^50^.
- The residential load represents 46% of the total consumed energy in Egypt^22^, and it exhibits an evening peak from 17:00 to 23:00 when solar generation is absent.
- Shifting a portion of the residential loads from evening to daytime hours would utilize the surplus solar generation, reduce the consumption of fossil fuels during the evening peak period, and improve the overall system efficiency.
- Starting from 2030, shifting 22% of the residential load from night to morning periods will present 10% of total demand (22% × 46% ≈ 10%). This percentage will cover the exact percentage of solar power generation surplus, which is 10% of total demand power, as revealed in the first aspect.

From 2030, the assumed scenario is investigated and publicized in Fig. 16 (b), in contrast to the unchanged load profile from 2024 to 2040 shown in Fig. 16 (a).

The impact of the EV home charging load will be investigated annually in the two scenarios: the base case scenario (unchanged load profile) and the second scenario, in which the load profile is updated to account for the DSM program to solar power integration. The simulation model output is added to the projection of the daily load curve to forecast the impact of the EV market penetration, along with the behavior of owners, on the load curve of the national grid, taking into consideration the five different scenarios resulting from the bass model forecast ($\:m$ = 10%, 20%, 30%, and 50%), and the two scenarios of load profile.

**Fig. 17:**
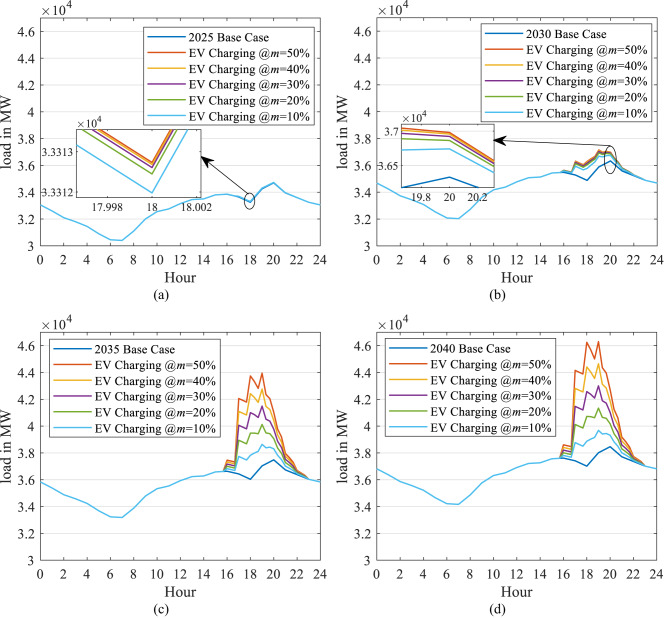
Impact of EV charging on the daily load curve for selected years (2025, 2030, 2035, 2040) under five market penetration scenarios ($\:m$=10%-50%), assuming an unchanged load profile shape.

A sample is displayed in Fig. 17 (a, b, c, and d) that comprises 2025, 2030, 2035, and 2040, respectively, in the case of the unchanged load profile. It reveals that the EV charging load increases the demand during peak hours considerably to higher values. In 2040, this new peak reaches 46 GW in the case of uncontrolled market penetration ($\:m$ =50%), while it reaches 39 GW in the case of controlled market penetration ($\:m$ =10%). This means that controlling the EV market penetration can save the installation of an extra 7 GW of generation capacity.

**Fig. 18:**
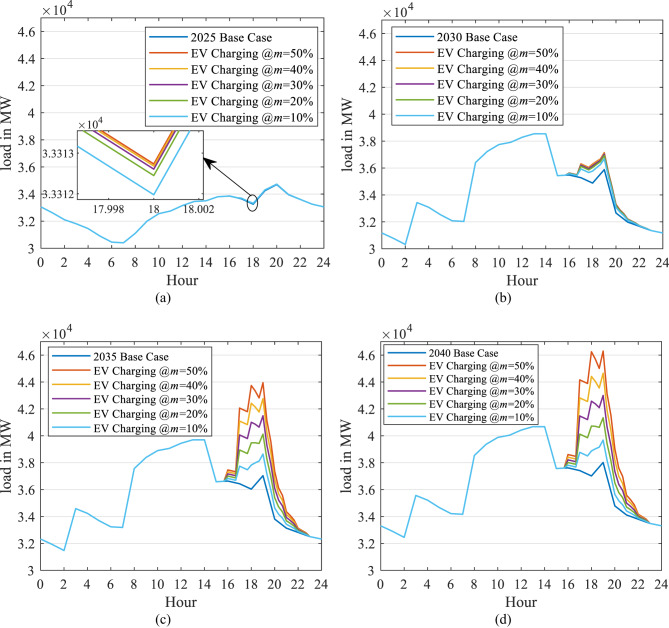
Impact of EV charging on the daily load curve for selected years under five market penetration scenarios, assuming an updated load profile shape from 2030 onwards due to DSM strategies and solar integration (shifting 22% of residential load from night to morning).

Figure 18 (a, b, c, and d) displays a sample of the results of uncontrolled EV home charging with the updated load profile in 2025, 2030, 2035, and 2040, respectively. It also comprises the five penetration scenarios ($\:m$ = 10%, 20%, 30%, and 50%) for each year.

Figures 17 (a) and 18 (a) reveal that the two load profile scenarios are identical in 2025 because the DSM application will commence in 2030. Consequently, the two scenarios differ from 2030 to 2040. In 2040, the updated load profile has the same peak in each of the five penetration scenarios. However, the load factor (LF) is higher compared to the case of an unchanged load profile. In 2040, in the event of uncontrolled market penetration ($\:m$ = 50%) and the updated load profile (Fig. 18 (d)), LF decreases by 9.5%, while for the case where the load profile remains unchanged, the LF is reduced by 14.34% (Fig. 17 (d)).

Similarly, in the case of controlled market penetration ($\:m$ =10%) with the updated load profile, the LF is improved by 0.62%, while in the case of an unchanged load profile, the LF is reduced by 2.46%. This occurs because of the shifted peak generated in the updated load profile. So, the new demand of the EV home charging added to the grid load profile improves the load profile performance.

### Evaluating peak load (PL) and load factor (LF)

**Fig. 19:**
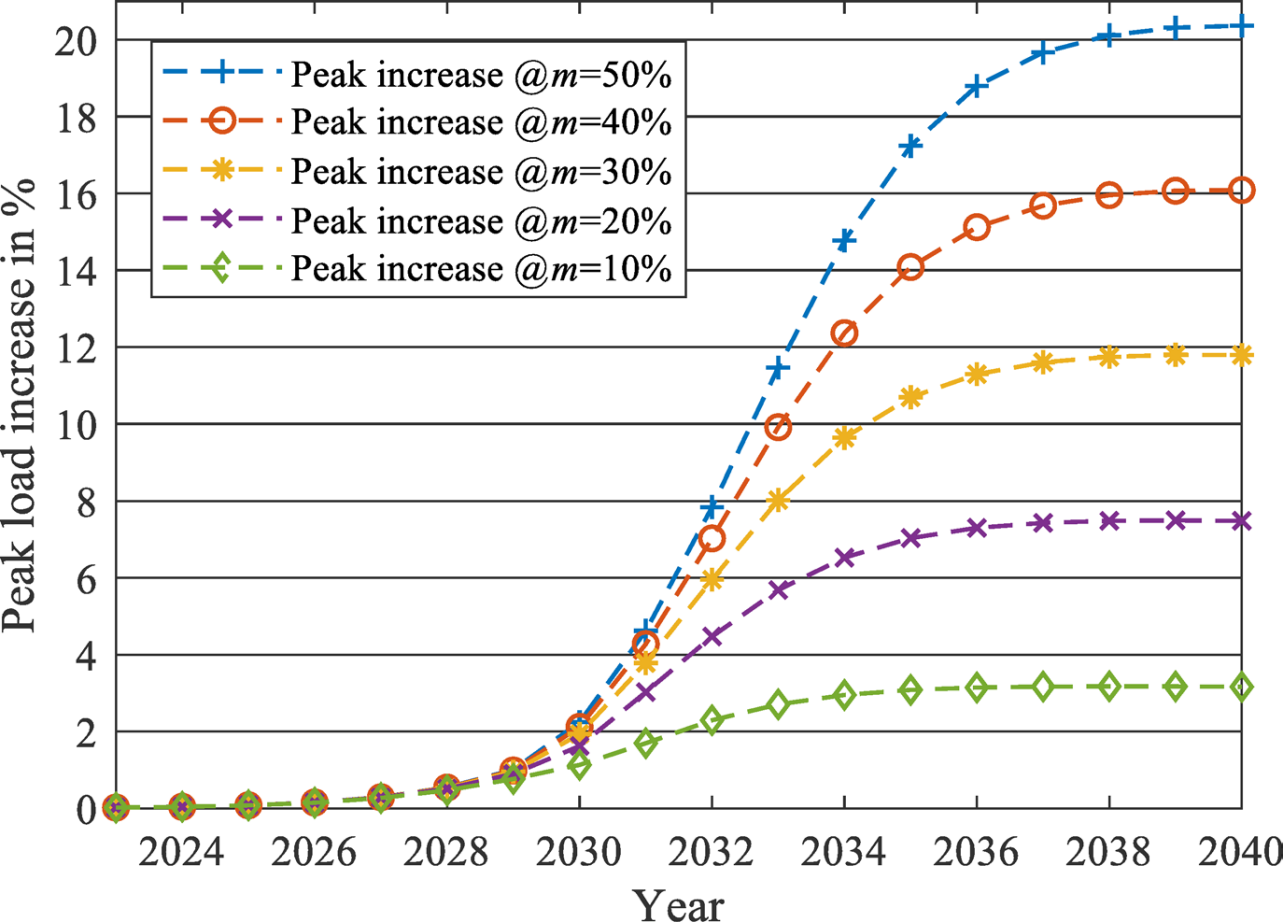
Percentage increase in PL due to the presence of EV charging load against the forecasted base load while neglecting the EV charging load at different $\:m$ values in case of unchanged load profile.

**Fig. 20:**
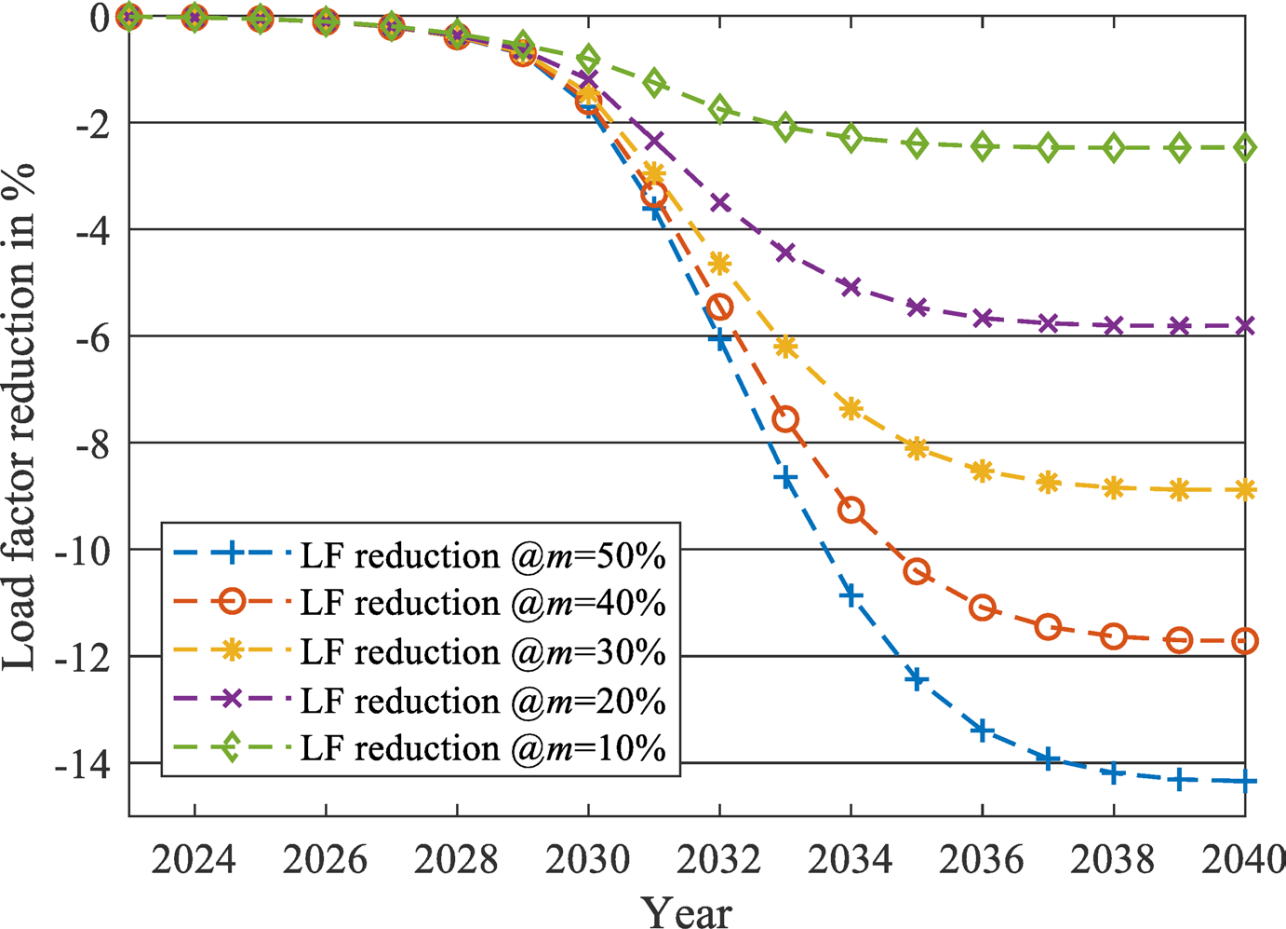
Percentage reduction in LF due to the presence of EV charging load against the forecasted base load while neglecting the EV charging load at different $\:m$ values in case of unchanged load profile.

**Fig. 21:**
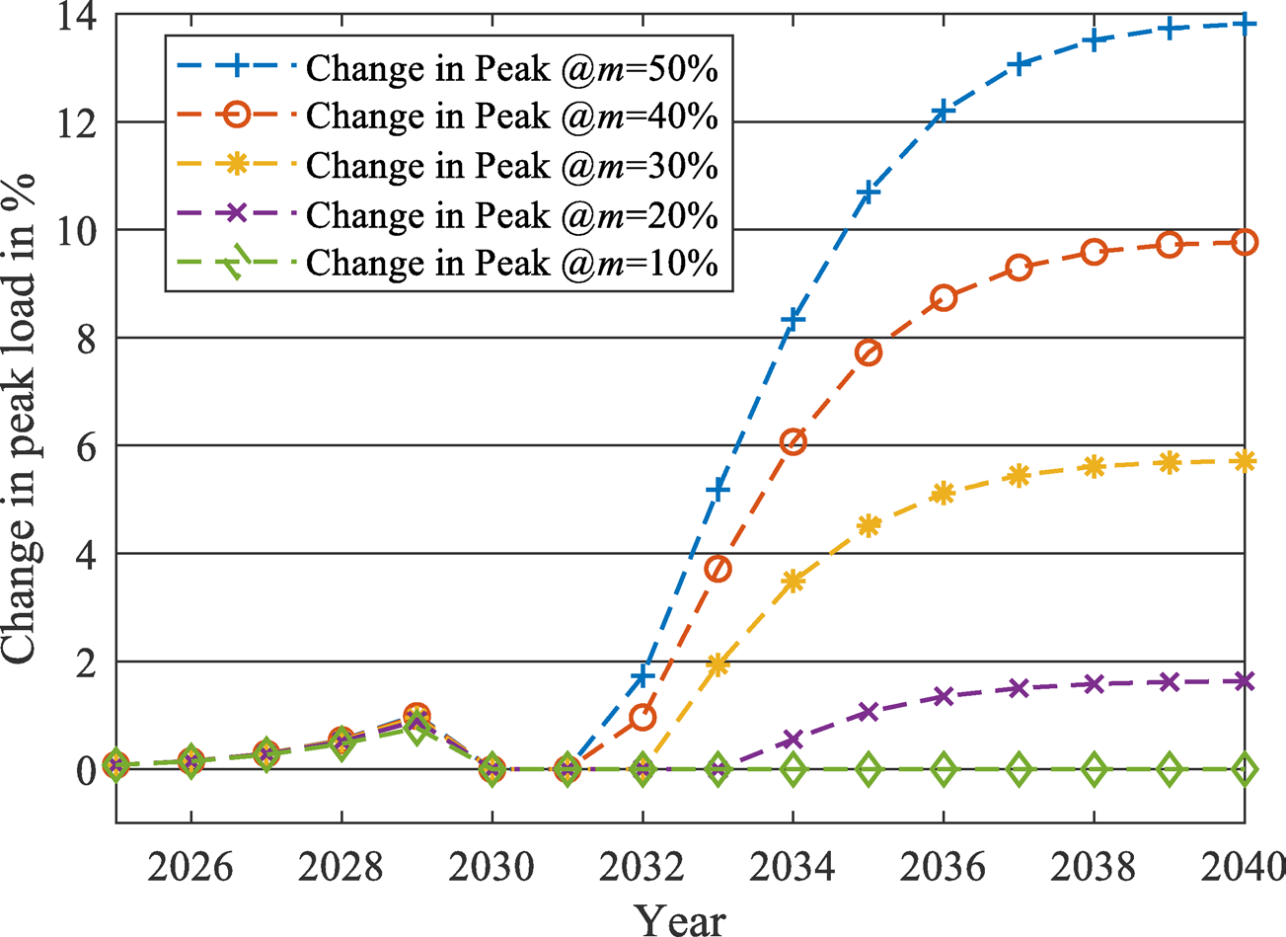
Percentage increase in PL due to the presence of EV charging load against the forecasted base load while neglecting the EV charging load at different $\:m$ values in case of updated load profile.

**Fig. 22:**
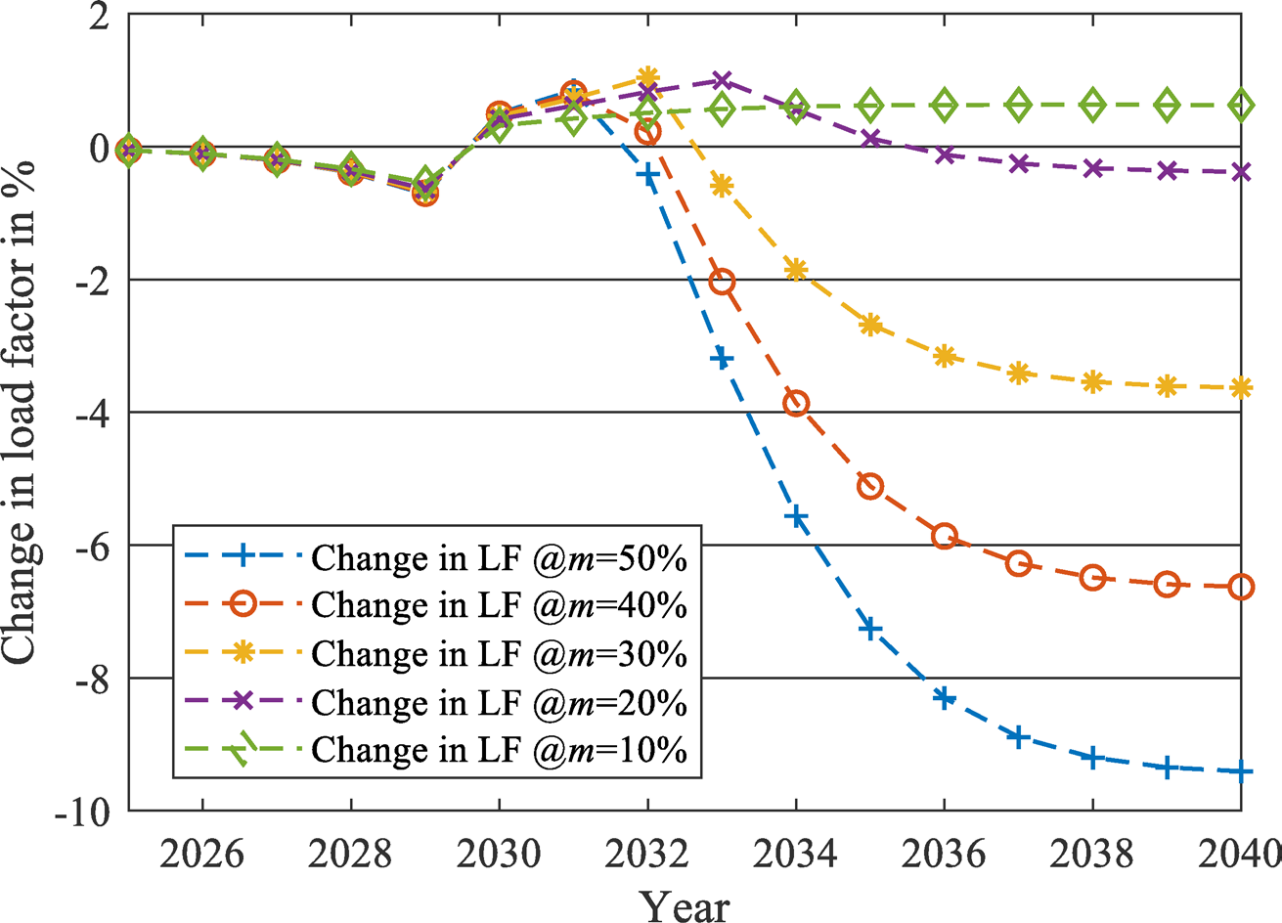
Percentage reduction in LF due to the presence of EV charging load against the forecasted base load while neglecting the EV charging load at different $\:m$ values in case of updated load profile.

After applying the proposed forecasting framework for each year from 2023 to 2040, the impact of the EV charging load on the two load profiles at each $\:m$ level is evaluated using two parameters: the percentage increase of peak load (PL) and the percentage reduction of LF. The percentages of changes from 2023 to 2040 are displayed in Figs. 19 and 20 for the unchanged load profile, while Figs. 21 and 22 are drawn for the updated load profile.

As revealed in Fig. 20, the LF will decrease exponentially at the early stages of EV market penetration until the EV market starts to reach its saturation phase. For example, at $\:m$=50%, the reduction saturates at -14.34% when the LF equals 0.8128 in 2040. The same pattern is also exposed to PL, as shown in Fig. 19. As the EV market starts to reach its saturation phase, the PL increase will saturate at 20.36% more than the base case predicted in 2040, to reach 46,298 MW PL in 2040 in the presence of the EV Market. Therefore, the LF and the PL will change similarly to the EV market penetration pattern (illustrated in Fig. 11), as discussed in the methodology section.

As displayed in Figs. 19 and 20, when $\:m$ is controlled to be 10% instead of 50%, the PL of the grid will only increase by 3.16% instead of 20.36% in 2040. Also, the LF will be reduced only by 2.46% instead of 14.34%.

On the other hand, Figs. 21 and 22 reveal the impact of EV home charging on the updated load profile when the DSM program is introduced to integrate solar power. The two figures show that the results start to be different from the unchanged load profile scenario starting from 2030, when the DSM is introduced to customers.

In the case of controlled EV penetration ($\:m$=10%), PL does not increase, and the LF improves by 2.46%, as the peak is shifted towards midday, while the introduced charging demand load is concentrated in the evening period, as discussed before. So, in that case, the charging demand load is considered as valley filling. This allows the grid to accommodate higher charging demand without a significant impact on the load profile. For example, in the case of $\:m$=20%, the PL does not increase till 2033, and LF improves by 0.99%. Compared to the unchanged load profile scenario, PL increases by 5.68% while LF reduces by 4.43%.

In the case of uncontrolled EV penetration ($\:m$=50%) and updated load profile, the impact of EV charging demand on the load profile is notably reduced. PL increases by 13.81%, which is less than the case of an unchanged load profile by 6.55%. Additionally, the LF is reduced by 9.4%, which is better than the case of an unchanged load profile by 4.94%.

After comparing the results in both scenarios (unchanged and updated load profiles), a critical finding is the mitigating effect of load profile management. As shown, introducing a DSM program provides a notable space for the grid to absorb the charging demand up to a certain level without impacting the load profile performance. Even in high penetration rates, the impact is notably reduced. This demonstrates that strategic grid planning can significantly alleviate the strain from high EV adoption.

### Possible policy implications

The results indicate that controlling the EV market penetration will strongly influence the impact of EV charging load on the load profile and, consequently, the grid generation performance. Therefore, adequate planning for the EV market penetration will influence utility planning significantly in terms of constructing new generation units or settling for the existing ones, depending on the available capacity. Moreover, introducing a DSM program will notably influence the impact. As shown in the previous section, shifting the native peak of the load profile to another period will give room for the grid to absorb the EV charging demand load to specific penetration rates without impacting the load profile performance. The following policy implications are recommended based on the results of this study:

- The government can increase the value of $\:m$ when the installed generation capacity is sufficient to absorb the new charging loads.
- The government can stifle the market to reduce the value of $\:m$ when the installed generation capacity does not cover the expected loads.
- Also, the government can plan to construct new generation capacities in parallel to the increasing market penetration to avoid power shortages or incur high initial costs.
- The government can start applying DSM programs to control the new EV charging load and utilize it to improve utility performance instead of overloading it. When such programs are implemented in parallel with growing market penetration, neither stifling the market nor constructing new generation units will be necessary^51^.

Controlling EV market penetration while planning for the electric utility will give the government a step ahead in avoiding power shortages or unsatisfactory performance.

### Limitations of the proposed framework

The proposed framework faces the following limitations while predicting the possible impact of the EV market penetration:

- The datasets for Egyptian energy demand in Sect. First stage: forecasting annual energy demand are limited because of the available statistical data. However, it was mitigated by applying several approaches in the energy demand forecasting process, considering the economic parameters, and validating the models using in-sample forecasting criteria to avoid overfitting.
- The sample size of the public survey may be small for presenting the EV fleet in upcoming years when the number of EVs reaches several million vehicles. It was due to the limited resources to get a larger sample size and the few statistical data available.
- Ignoring other EV fleet categories, such as official EVs, taxis, and buses, because of the minimal presence of such categories in the Egyptian market at the moment.
- Assuming that the behavior of conventional vehicle owners provides a valid proxy for future EV owners, due to the recent limited number of EV owners in Egypt.

The key factor to mitigate these limitations is to update the framework’s inputs periodically to enhance its forecasting capabilities and increase the sample size of the public survey, ensuring that the probabilistic model can sufficiently represent the entire fleet.

## Conclusions and future work

This study presents a scalable framework for long-term forecasting of EV charging impacts on the national grid, integrating projections for baseline energy demand, EV market penetration scenarios, and user charging behavior. The framework was applied to Egypt to provide clear guidelines for policymakers to forecast the impact of uncontrolled EV market penetration on a long-term basis. It can be applied to a specific area, a city, or a country. The framework starts by predicting the energy demand, then forecasting the EV market penetration rate, and including the uncertainties due to the stochastic behavior of EV owners, such as the plug-in time, plug-in rate at home, and the duration of charging. Indeed, the framework will provide more accurate predictions when the developed models are updated over time on a year-by-year basis.

The designed framework is applied in this study to the Egyptian market. The results revealed that when the EV market penetration is not controlled, whether by policies or marketing strategies, a fast adoption of EVs will occur and affect the utility grid considerably. Based on the forecasted results till 2040, when the expected adopters are 50% of the total market, the utility PL may reach 20.36% more than the predicted demand when excluding EV charging load. Furthermore, the LF may decrease by 14.34% lower to the projected demand in the case of excluding EV charging load. On the other hand, when the EV market is controlled, and the expected number of adopters is only 10% of the total market, the peak demand is increased by only 3.16% more than the predicted demand without the EV charging load. Furthermore, the LF may be decreased by only 2.46% lower to the case of predicting demand without the EV charging load.

The robustness of the framework is verified by assuming a scenario in which the load profile shape is changed due to the application of a DSM program to integrate high solar power generation. The DSM program shifts 22% of the residential load from the night period to the morning period. It shifts the native peak of the original load profile from the evening period to midday. Then the same analysis is done on the updated load profile. The results revealed that the impact of the EV home charging demand is notably reduced. For example, when the expected adopters are 50%, the PL increases by 13.81% which is less than the case of an unchanged load profile by 6.55%. Also, the LF is reduced by 9.4% which is better than the case of an unchanged load profile by 4.94%. On the other hand, when the expected adopters are 10%, PL does not increase, and the LF improves by 2.46%.

Therefore, policymakers should take vital actions, either by controlling the market penetration rate or by managing the charging process using one of the DSM programs to avoid undesirable utility circumstances.

As part of future work, the proposed framework can be extended by investigating the impact of different EV fleets (such as officials, taxis, and buses), taking into consideration the public charging stations, and increasing the resolution of the framework by applying it to smaller sections of the grid and integrating the results. Moreover, the impact of several DSM programs that can be applied in Egypt, such as load shifting, vehicle-to-grid (V2G), and centralized charging optimization for mitigating the negative consequences of EV charging, could be extensively investigated and assessed.

## Data Availability

All data generated or analyzed during this study are included in the article. The questionnaire in Arabic will be made available upon request.
